# Cuban *Calisto* (Lepidoptera, Nymphalidae, Satyrinae), a review based on morphological and DNA data

**DOI:** 10.3897/zookeys.165.2206

**Published:** 2012-01-13

**Authors:** Rayner Núñez Aguila, Edelquis Oliva Plasencia, Pavel F. Matos Maravi, Niklas Wahlberg

**Affiliations:** 1 División de Colecciones Zoológicas y Sistemática, Instituto de Ecología y Sistemática, Carretera de Varona km 3.5, Capdevila, Boyeros, Ciudad de La Habana, Cuba; 2 Laboratory of Genetics, Department of Biology, University of Turku, FI–20014 Turku, Finland

**Keywords:** Taxonomy, speciation, DNA, habitat, distribution, life cycle, immature stages, Greater Antilles

## Abstract

The Cuban species of *Calisto* are reviewed based on the morphology of adult and immature stages, as well as DNA sequences of six genes (COI, EF1α, *wingless*, GAPDH, RpS5, CAD). A new species, *Calisto occulta* **sp. n.**, is described from the northeastern Cuban mountains. *Calisto smintheus* Bates, 1935 and *Calisto bruneri*, Michener 1949 are revised and revalidated. A new status, the species level, is proposed for *Calisto brochei*, Torre 1973, *Calisto muripetens*, Bates 1939 and *Calisto bradleyi*, Munroe 1950. The immature stages of *Calisto smintheus*, *Calisto brochei*,and *Calisto occulta* are described for the first time, and those of *Calisto herophile*, Hübner 1823 are redescribed. Useful morphological characters for adults are the shape and conspicuousness of androconial patch, the number and relative size of white dots on underside of hindwing, the shape of aedeagus, the shape of digitiform projection of genitalia valve, the shape and relative size of tegumen and uncus, the relative size of female genitalia, the height of sterigmal ring dorsal crown of the latter, and the relative size of corpus bursae and ductus bursae. For the immature stages, the most important characters are the color pattern of head capsule, the number and width of longitudinal lines of body, in the larvae; and the color pattern and the absence or presence of dorsal ridges on the abdomen of pupae. The phylogenetic relationships between the Cuban *Calisto* species are quite robust and well-supported; however, conflict between mitochondrial and nuclear datasets was detected in *Calisto brochei*, *Calisto muripetens* and to a lesser degree in *Calisto bradleyi*.

## Introduction

The genus *Calisto* Hübner, 1823 is endemic to the West Indies and is the only representative of the subfamily Satyrinae (Nymphalidae) in the area. Lamas (2004) listed 42 *Calisto* species, 37 of them from Hispaniola and the remainder present on Cuba, Jamaica, Puerto Rico, Anegada Island and Bahamas. Until late the 1960s, the taxonomy of the Cuban species was relatively stable with most of the original names retained after several works (Bates 1935; 1939; Clench 1943; Michener 1949; Munroe 1950; Torre 1952, 1954, 1968). However, Brown and Heineman (1972) treated all Cuban species as *Calisto herophile* Hübner, 1823 and *Calisto sibylla* Bates, 1934 without giving any taxonomic reason, a decision criticized soon by Munroe (1972). The majority of subsequent authors (Alayo and Hernández 1987; Smith et al. 1994; Lamas 2004) have maintained this unjustified treatment. Núñez (2009) supported the use of original names until an in-depth review of the Cuban and Bahamian species takes place.

Several factors delayed the clarification of Cuban *Calisto* taxonomy, of which the most important is the cryptic nature of most species, with adults showing little morphological differences. Also, some of the few useful adult characters have received poor attention by researchers, *e.g.* shape and conspicuousness of androconial patch, the structure of male and female genitalia; whereas others have been overused or misused, *e.g.* shape of red spot at underside of forewing cell, number and relative size of white dots at underside of hindwing. Characters of immature stages of most species remained unavailable until the present work. They have proven to be useful in the taxonomy of Hispaniolan members of genus (Sourakov and Emmel 1995, Sourakov 1996, 1999).

In the present work, we review the Cuban species of *Calisto* and describe a new species from the northeastern Cuban mountains. Several taxonomic changes based on both morphological and molecular evidence are proposed. Detailed diagnoses are provided for each species. The male and female genitalia of all Cuban species are fully illustrated and described. A key for all species known from Cuba is also provided. Natural history notes, including new localities, habitat, nectar sources, and description of immature stages, are compiled for all Cuban *Calisto*. DNA sequencing is used here for the first time in the taxonomy of Cuban *Calisto*. Only *Calisto herophile* was included recently in a DNA barcoding study involving the Hispaniolan, Jamaican and Puerto Rican species of *Calisto* (Sourakov and Zakharov 2011). Here we sequenced six molecular markers, one mitochondrial (COI) and five nuclear genes (EF1α, wingless, GAPDH, RpS5 and CAD), in order to clarify the status and relationships of all known Cuban taxa.

## Materials and methods

### Collection and rearing of immature stages

Eggs were obtained by confining females to plastic jars of 5 oz. After being laid, the eggs remained untouched (no measures were taken) until larvae hatched. Egg collection data: *Calisto herophile*– Pinar del Río, Sierra del Rosario, Rangel, 19–20 April 2009, *Calisto occulta*– Holguín, Moa, Yamanigüey, 25 September 2009, *Calisto smintheus smintheus* – Santiago de Cuba, Gran Piedra, near Estación BIECO, 25 February 2011, *Calisto smintheus brochei* – Guantánamo, Baracoa, northern slope of Monte Iberia, 3–4 May 2011. Larvae were maintained at ambient temperature, humidity and photoperiod in Havana. For all species, two introduced common grass species, *Zoysia japonica* and *Cynodon dactylon*, were used daily as substitute host plants. Width and height of head capsules and length of larvae, at first instar, were measured with an ocular micrometer having 0.01 mm of precision mounted in a Carl Zeiss Stemi 2000 stereoscopic microscope. Length of last instar larvae and pupae were measured with a metric ruler of 1 mm of precision.

### Dissections, characters & descriptions

Wings were cleared with sodium hypochlorite, Eosin–Y tinged and mounted in Euparal. Genitalia and other body parts were treated with hot 10% potassium hydroxide (KOH) solution and the cleaned material was stored in glycerine.

Morphological characters for adults were those traditionally used in previous studies on *Calisto*. For wing pattern, we follow Smith et al. (1994), Jonhson and Hedges (1998), and Núñez (2009). For male genitalia, we follow the terms used by Núñez (2009) and for the female genitalia those detailed by Johnson et al. (1987). Species descriptions and the key are based primarily on fresh specimens. Recently collected individuals show all details of color pattern, mainly those important on the under surface of the wings, which fade relatively fast after death (Smith et al. 1994; Sourakov in Johnson and Hedges 1998). For immature stages, the characters given by Sourakov and Emmel (1995) and Sourakov (1996, 1999) were used. The nomenclature for the longitudinal lines of larvae was after Dethier (1940) except for the para–dorsal for which case the subdorsal line was used.

## DNA analysis

Two butterfly legs per individual were preserved either desiccated or immersed in ethanol. Total DNA was extracted from legs using the DNEasy extraction kit (QIAGEN). Six molecular markers including one mitochondrial (COI) and five nuclear genes (EF1α, wingless, GAPDH, RpS5 and CAD) were amplified using previously published primers and protocols (Wahlberg and Wheat 2008). DNA sequencing was carried out by the company Macrogen-South Korea. Sequence editing and alignment were done manually in the program BioEdit v7.0.5 (Hall 1999). Voucher photos are available at the Nymphalidae Systematics Group database (http://nymphalidae.utu.fi/db.php) and DNA sequences have been submitted to GenBank (Annex 1).

**Annex 1.** GenBank accession numbers to sequenced genes of Cuban and Hispaniolan (outgroup) *Calisto* specimens used in present study.

	**Species**	**Voucher code**	**Collection locality**	**coi**	**ef1a**	**wingless**	**gapdh**	**rps5**	**cad**
Outgroup	*Calisto arcas*	NW149-16	DOMINICAN REPUBLIC: La Vega Province, 22 km SE of Costanza, Hwy 41	JN881877	JN881759	JN881855	JN881811	JN881829	JN881779
	*Calisto chrysaoros*	DR017	DOMINICAN REPUBLIC: La Ciénaga, La Vega	JN881878	JN881760	JN881856	JN881812	JN881830	JN881780
	*Calisto confusa*	DR016	DOMINICAN REPUBLIC: La Ciénaga, La Vega	JN881879	JN881761	JN881857	JN881813	JN881831	JN881781
	*Calisto obscura*	DR080	DOMINICAN REPUBLIC: Boca de Yuma, Parque Nac. del Este	JN881880	JN881762	JN881858	JN881814	JN881832	JN881782
	*Calisto pulchella*	DR003	DOMINICAN REPUBLIC: Puerto Plata	GQ357225	GQ357292	GQ357357	GQ357467	GQ357596	JN881783
	*Calisto bradleyi*	PM07-06	CUBA: El Taburete, Sierra del Rosario	JN881881	JN881763	JN881859	JN881815	JN881833	JN881784
	*Calisto bradleyi*	PM07-24	CUBA: Base norte Mogote Dos Hermanas	JN881882	JN881764	JN881860	JN881816	JN881834	JN881785
	*Calisto bradleyi*	PM07-25	CUBA: Base norte Mogote Dos Hermanas	JN881883	-	JN881861	JN881817	JN881835	JN881786
	*Calisto bradleyi*	PM07-26	CUBA: Base norte Mogote Dos Hermanas	JN881884	JN881765	JN881862	JN881818	JN881836	JN881787
	*Calisto brochei*	PM07-03	CUBA: Ladera norte Monte Iberia, cerca de antiguo campamento minero	JN881885	JN881766	JN881854	-	JN881828	JN881788
	*Calisto brochei*	PM15-03	CUBA: South of Tetas de Julia, Monte Iberia	JN881871	-	-	-	-	-
	*Calisto brochei*	PM07-20	CUBA: Estación La Zoilita	JN881886	JN881767	JN881863	JN881819	JN881837	JN881789
	*Calisto bruneri*	PM07-15	CUBA: Cayo Grande, Moa	JN881887	JN881768	JN881846	-	-	JN881790
	*Calisto bruneri*	PM07-16	CUBA: Cayo Grande, Moa	JN881888	JN881769	JN881847	-	-	JN881791
	*Calisto bruneri*	PM07-17	CUBA: Yamanigüey	JN881889	-	-	-	-	JN881792
	*Calisto bruneri*	PM07-21	CUBA: Estación La Zoilita	JN881890	JN881770	JN881864	JN881820	JN881838	JN881793
	*Calisto herophile apollinis*	PM13-01	BAHAMAS: New Providence I., Prospect Ridge Natl. Pk.	JN881872	-	-	-	-	-
	*Calisto herophile apollinis*	PM13-02	BAHAMAS: New Providence I., Prospect Ridge Natl. Pk.	JN881873	-	-	-	-	-
	*Calisto herophile herophile*	CP19-16	CUBA: La Habana	JN881874	-	-	-	-	-
	*Calisto herophile herophile*	PM07-07	CUBA: Río Guajaibón, La Habana	JN881891	-	-	-	-	JN881794
	*Calisto herophile herophile*	PM07-12	CUBA: Loma del Gato, Sierra del Cobre	JN881892	-	JN881848	-	-	JN881795
	*Calisto herophile herophile*	PM07-22	CUBA: Estación La Zoilita	JN881893	JN881771	JN881865	JN881821	JN881839	JN881796
	*Calisto herophile herophile*	PM15-06	CUBA: Camino de La Melba, Moa	JN881894	-	JN881849	-	-	JN881797
	*Calisto israeli*	PM07-01	CUBA: Ladera norte Monte Iberia, cerca de antiguo campamento minero	JN881895	JN881772	JN881866	JN881822	JN881840	JN881798
	*Calisto israeli*	PM07-02	CUBA: Morones, cerca de La Melba	JN881896	JN881773	JN881867	JN881823	JN881841	JN881799
	*Calisto israeli*	PM07-27	CUBA: Antiguo campamento minero Meseta de El Toldo	JN881875	-	-	-	-	-
	*Calisto muripetens*	PM07-08	CUBA: Carso de Buenos Aires	JN881897	-	-	-	-	JN881800
	*Calisto muripetens*	PM07-11	CUBA: Carso de Buenos Aires	JN881898	-	JN881868	JN881824	JN881842	JN881801
	*Calisto muripetens*	PM15-02	CUBA: Pico San Juan	JN881876	-	-	-	-	-
	*Calisto occulta*	PM07-04	CUBA: Tetas de Julia	JN881899	JN881774	-	JN881825	JN881843	JN881802
	*Calisto occulta*	PM07-10	CUBA: Tetas de Julia	JN881900	-	-	-	-	JN881803
	*Calisto occulta*	PM07-18	CUBA: Yamanigüey	JN881901	JN881775	JN881869	JN881826	JN881844	JN881804
	*Calisto occulta*	PM07-19	CUBA: Yamanigüey	JN881902	JN881776	JN881850	-	-	JN881805
	*Calisto occulta*	PM07-23	CUBA: Yamanigüey	JN881903	JN881777	JN881851	-	-	JN881806
	*Calisto smintheus*	PM07-05	CUBA: Alrededores de La Platica	JN881904	JN881778	JN881870	JN881827	JN881845	JN881807
	*Calisto smintheus*	PM07-09	CUBA: Ladera sur Pico Regino	JN881905	-	-	-	-	JN881808
	*Calisto smintheus*	PM07-13	CUBA: Loma del Gato, Sierra del Cobre	JN881906	-	JN881852	-	-	JN881809
	*Calisto smintheus*	PM07-14	CUBA: Loma del Gato, Sierra del Cobre	JN881907	-	JN881853	-	-	JN881810

Genetic distances were calculated using the program MEGA4 (Tamura et al. 2007) using the Kimura 2-Parameter model and the Pairwise Distance Calculation analysis for the partial sequence of COI gene, following the DNA barcoding approach (Hebert et al. 2003; Hebert et al. 2004). Phylogenetic analyses were carried out in the program MrBayes v3.1 (Ronquist and Huelsenbeck 2003) and executed through the CIPRES web portal (http://www.phylo.org/sub_sections/portal/). The data were partitioned by gene and analyzed as independent partitions. Due to conflicting results, we chose to analyze the mitochondrial and nuclear genes separately. We imposed the GTR+G sequence evolution model to every partition based on the Log Likelihood values and the Akaike Information Criteria (AIC) calculated using the FindModel portal (http://www.hiv.lanl.gov/content/sequence/findmodel/findmodel.html). Two independent MCMC analyses with four simultaneous chains (one cold and three heated) on each analysis were run for 10 million generations and the sampling of trees was set to every 1000 generations. Convergence of the two runs was determined by the stationary distribution plot of the log likelihood values against number of generations and confirmed by the average standard deviation of split frequencies which in all the cases were lower than 0.05. We discarded the first 1000000 generations as burn-in and trees were summarized under the 50 percent majority rule method.

### Repository abbreviations

AMNH American Museum of Natural History, New York, USA.

CZACC Instituto de Ecología y Sistemática, Havana, Cuba.

CMNH Carnegie Museum of Natural History, Pittsburgh, USA

CUIC Cornell University Insect Collection, Ithaca, USA

MFP Museo Felipe Poey, Havana, Cuba.

FZC Private collection of Fernando de Zayas, Havana, Cuba.

MCZ Museum of Comparative Zoology of Harvard, Cambridge, MS, USA

Type material of Cuban and Bahaman *Calisto* deposited at Museum of Comparative Zoology, Harvard, except *Calisto smintheus muripetens* Bates 1939, was reviewed through pictures available on Internet by the E–Type Initiative (Perkins et al. 2005).

### Other abbreviations

dl discal line

FW forewing

HW hindwing

NSB Nipe–Sagua–Baracoa

pdl postdiscal line

stl subterminal line(s)

M1–M2 interspace between median (M) veins 1 and 2

M2–M3 interspace between median (M) veins 2 and 3

M3–Cu1 interspace between median (M) vein 3 and cubital (Cu) vein 1

Rs–M1 interspace between Radial sector (Rs) and median (M) vein 1

UN under side

UP upper side

UNFW under side of forewing

UNHW under side of hindwing

UPFW upper side of forewing

UPHW upper side of hindwing

## Results

### Calisto israeli

Torre, 1973

http://species-id.net/wiki/Calisto_israeli

**Figures 1–12.**
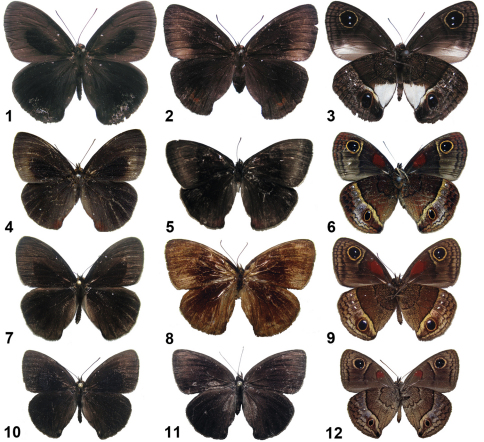
Cuban *Calisto* adults. **1** *Calisto israeli* ♂upper side, Guantánamo, Baracoa, Monte Iberia, al sur de las Tetas de Julia **2** *Calisto israeli* ♀upper side, same locality **3** *Calisto israeli* ♂under side **4** *Calisto smintheus* ♂upper side, Granma, Sierra Maestra, La Platica **5** *Calisto smintheus* ♀upper side, Granma, Bartolomé Masó, ladera sur Pico Regino **6** *Calisto smintheus* ♂under side **7** *Calisto brochei* ♂upper side, Guantánamo, Baracoa, ladera norte Monte Iberia **8** *Calisto brochei* paratype ♀upper side, Oriente (currently Guantánamo), Cupeyal **9** *Calisto brochei* ♂under side **10** *Calisto bruneri* ♂upper side, Holguín, Sierra de Cristal, cerca de Estación La Zoilita 400 m. **11** *Calisto bruneri* ♀upper side, Holguín, Moa, Cayo Grande **12** *Calisto bruneri* ♂under side.

Figs 1–3 25 32 40 48 56, 57 60–62 66

Calisto israeli Torre 1973: 3, Alayo and Hernández 1987: 41, Núñez 2009: 49
Calisto israel Smith et al. 1994: 57, misspelling
Calisto sibylla smintheus Lamas 2004: 207

#### Diagnosis.

*Calisto israeli* can be separated from all its congeners by the large, triangle shaped patch of white scales at the middle portion of the inner margin at UNHW.

**Description.** FWL: 24–26 mm ♂, 25–27 mm ♀. Male UPFW uniform brown except basal two thirds of costa and androconial patch, dark brown almost black (Fig. 1). Androconial patch extending diagonally between posterior margin of cell and 2A vein to beyond M_3_ origin, outer and posterior margins rounded, about three fifths the length of FW (Fig. 32). Female UPFW with basal three fifths and outer margin dark brown, outer two fifths pale brown (Fig. 2). Male UPHW uniform dark brown, costa pale brown. Female UPHW dark brown at anterior two thirds, posterior third pale brown. UN of wings brown mixed with ochre and, in less extent, pale yellow scales mostly at basal half (Figs 3, 25); interspace of stl pale brown mixed with pale yellow scales. UN lines of wings without external edge of pale scaling, only white scales on outer edge of pdl at posterior half of wing. UNFW without red on cell and white scaling below cell to posterior margin. Post discal area on UNHW with four white dots at Rs–M_1_, M_1_–M_2_, M_2_–M_3_, dot at M3–Cu_1_ if present very small; middle of UNHW posterior margin with a large triangular patch of white scales; post discal area heavily suffused with white scales. HW anal lobe entirely black at UN. Male genitalia heavily sclerotized, tegumen approximately 0.7 the length of uncus, with dorsum nearly flat (Fig. 40); uncus stout and slightly arched, tapering gradually to apex, base slightly protruding, subquadrate; digitiform projection of valve straight with ventral margin slightly concave; aedeagus swollen at base in lateral view, near straight with a small left curve at basal half in dorsal view. Female genitalia large (Fig. 48); dorsal crown very tall; corpus bursae broad, approximately 0.8 the length of ductus bursae.

#### Type material.

Holotype♀: Oriente (currently Guantánamo), Cupeyal 730 m, 20°26'57"N, 75°03'38"W, V/1971, I. García. CZACC, examined. Paratypes 2 ♂, 5 ♀: same data as for holotype except VI/1971, genitalia ♀ in glycerin. CZACC, MFP, examined.

#### Additional material.

10 ♂, 6 ♀. **Holguín:** Morones, cerca de La Melba 250 m, 20°26'22"N, 74°49'14"W, 22/V/2007, N. Fernández, genitalia in glycerin, DNA voucher PM07–02 (M002) (1 ♂); antiguo campamento minero Meseta de El Toldo 800 m, 20°27'20"N, 74°54'02"W, IV/2008, E. Pérez, genitalia in glycerin, DNA voucher PM07–27 (M046) (3 ♂). **Guantánamo:** Baracoa, Monte Iberia, campamento ladera norte 600 m, 20°29'25.5"N, 74°43'51.3"W, 18/V/2007, R. Núñez, slide RNA162(wings), DNA voucher PM07–01 (M001) (1 ♂); Baracoa, Monte Iberia, ladera sur cerca de la cima 675 m, 20°27'23.9"N, 74°44'27.9"W, 20/V/2007, R. Núñez, genitalia in glycerin, slides RNA170 (legs & labial palpus)/171(wings) (2 ♂); Baracoa, Monte Iberia, Tetas de Julia 650 m, 20°27'47"N, 74°45'13.3"W, 20/V/2007, R. Núñez, genitalia ♂ in glycerin, slides RNA160/164 (wings)/172/173 (legs & labial palpus)/176(androconial scales) (2 ♂, 2 ♀); Baracoa, Monte Iberia, al sur de las Tetas de Julia 430 m, 20°27'47"N, 74°45'13.3"W, 20/V/2007, R. Núñez, slides RNA168(legs & labial palpus) (1 ♂, 1 ♀); Baracoa, Monte Iberia, ladera norte 385 m, 20°29'53"N, 74°43'48"W, 1/V/2011, R. Núñez (3 ♀). CZACC.

#### Distribution.

Collected specimens of *Calisto israeli* come from several localities in the middle and western parts of the NSB mountains, from Monte Iberia plateau 25 km west to Cupeyal (Figs 56, 57). The species has also been recorded from Sierra de Cristal, 1230 m, during the last management plan of Pico Cristal National Park (ENPFF 2010), extending its known distribution almost 50 km west compared to previous records. Species is probably present on the eastern half of NSB whenever its habitat is preserved.

#### Immature stages.

 Eggs are laid loose, are near spherical in shape and ivory white in color.

#### Habitat and biology.

 The species inhabits several variants of evergreen and rainforests and, to a lesser extent, scrub forests (charrascales) of the NSB Mountains at altitudes between 250 and 1230 m (Figs 60–62). Individuals can be found mainly on forest paths and clearings both sunny and shady. Núñez (2009) recorded 28 individuals along 1.5 km of old mining roads. At Pico Cristal, during an ascent from the foothills to the top, 356 individuals of this species were recorded (ENPFF 2010). Althoughits life story is unknown, the species seems to be associated with climbing grasses like some of its Hispaniolan congeners (Smith et al. 1994; Schawrtz and Wetherbee 1996). In different visits to Monte Iberia plateau, the senior author found the species abundant only at sites where two climbing grass species, *Arthrostylidium pinifolia* and *Chusquea* sp., dominated the lower strata of the rainforest (Fig. 61). The only mating pair observed was found on May 2011, 3:30 pm, at one of the sites covered by grasses mentioned above.

#### Remarks.

The distinctive pattern of *Calisto israeli* permits a straightforward separation of the species from all its congeners, mostly based on a white triangular patch on the middle posterior margin at UNHW and the lack of red in cell at UNFW. Nuclear DNA analysis grouped *Calisto israeli* alongwith *Calisto smintheus* and *Calisto brochei* in a branch separated from the remainder of the Cuban taxa (Fig. 66), although the mitochondrial COI dataset suggests an earlier branching event of the *Calisto israeli* lineage in the phylogeny, placing it as sister to the rest of Cuban *Calisto*. Moreover, the genetic distances regarding the COI sequence support the recognition of *Calisto israeli* as a valid species since the minimum distance to the closely related *Calisto smintheus* is 9.01% while the average divergence percentages from other congeneric species is higher than 5%.

**Figures 13–24.**
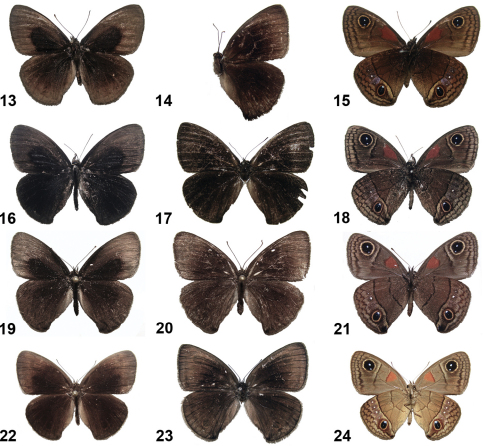
Cuban *Calisto* adults. **13** *Calisto muripetens* ♂upper side, Cienfuegos, Pico San Juan **14** *Calisto muripetens* ♀upper side, Sancti Spiritus, Topes de Collantes **15** *Calisto muripetens* ♂under side **16** *Calisto occulta*, new species,holotype ♂upper side, Guantánamo, Baracoa, Monte Iberia plateau, Tetas de Julia **17** *Calisto occulta*, new species,paratype♀upper side, same locality **18** *Calisto occulta*, new species,holotype ♂under side **19** *Calisto bradleyi* ♂upper side, Pinar del Río, base norte mogote Dos Hermanas **20** *Calisto bradleyi* ♀upper side, same locality **21** *Calisto bradleyi* ♂under side **22** *Calisto herophile* ♂upper side, Matanzas, Varadero, Varahicacos **23** *Calisto herophile* ♀upper side, Artemisa, Sierra del Rosario, El Taburete **24** *Calisto herophile* ♂under side.

**Figures 25–31.**
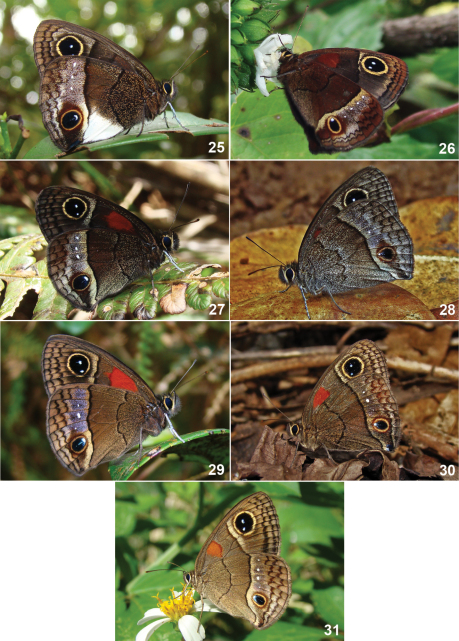
Live adults of Cuban *Calisto*. **25** *Calisto israeli* **26** *Calisto smintheus* **27** *Calisto brochei* **28** *Calisto bruneri* **29** *Calisto occulta*, new species **30** *Calisto bradleyi* **31** *Calisto herophile*.

**Figures 32–39.**
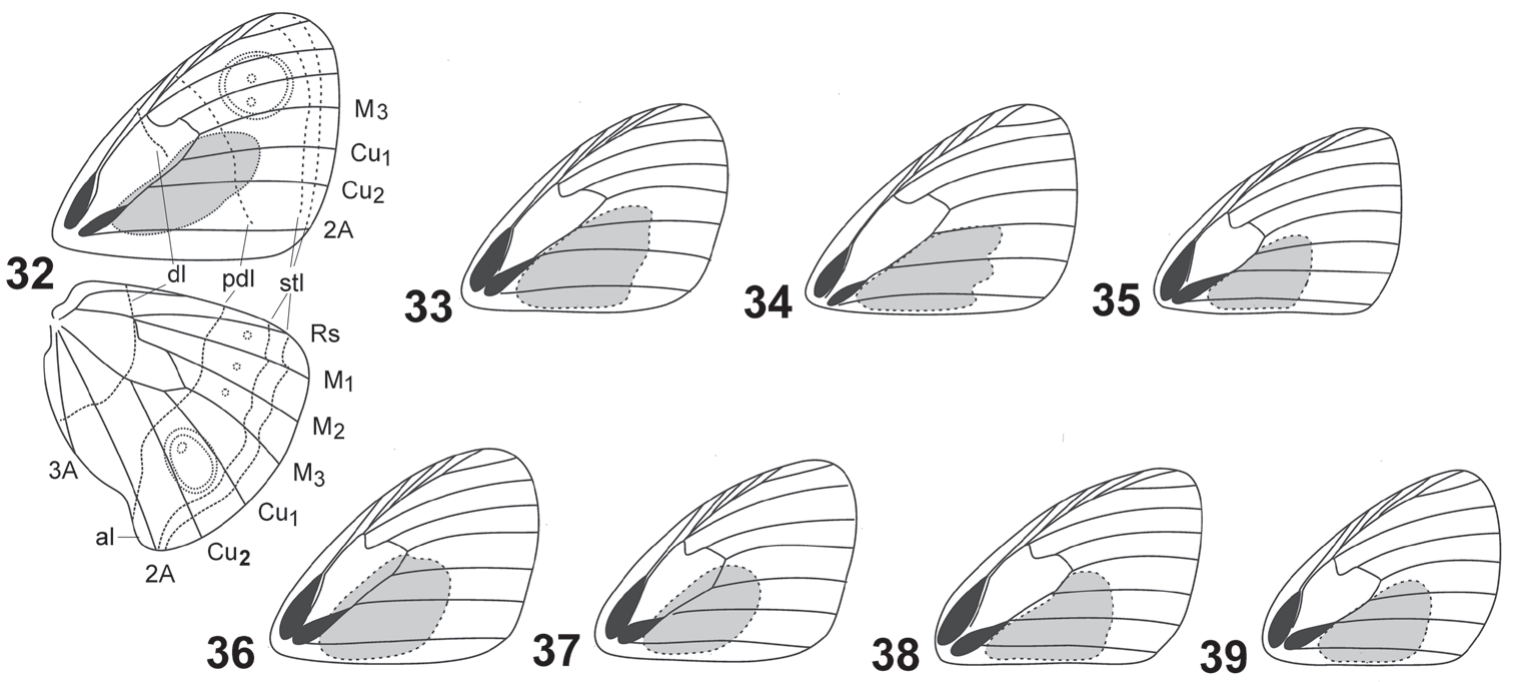
Shape and location of androconial patch, under side lines, ocelli, white dots and realted veins in Cuban species of *Calisto*. **32** *Calisto israeli* **33** *Calisto smintheus* **34** *Calisto brochei* **35** *Calisto bruneri* **36** *Calisto muripetens* **37** *Calisto occulta*, new species **38** *Calisto bradleyi* **39** *Calisto herophile* Abbreviations: al– anal lobe, dl– discal line, pdl– postdiscal line, stl– subterminal lines, M_1_– median vein 1, M_2_– median vein 2, M_3_– median vein 3, Cu_1_– cubital vein 1, Cu_2_– cubital vein 2, Rs– radial sector.

**Figures 40–47.**
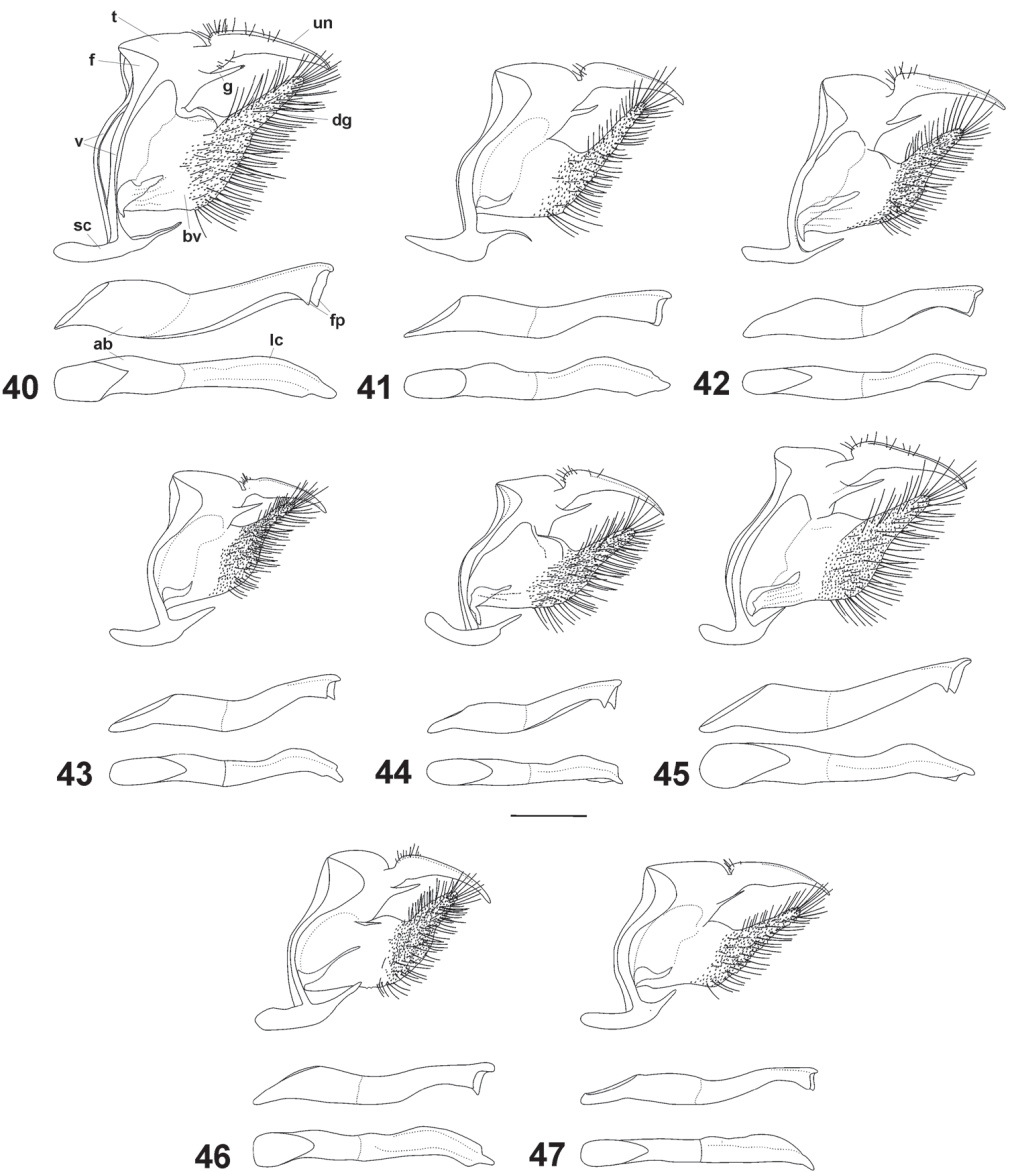
Male genitalia of Cuban *Calisto* from top to bottom: main body in lateral view aedeagus in lateral view and aedeagus in dorsal view. **40** *Calisto israeli* **41** *Calisto smintheus* **42** *Calisto brochei* **43** *Calisto bruneri* **44** *Calisto muripetens* **45** *Calisto occulta* new species **46** *Calisto bradleyi* **47** *Calisto herophile* Abbreviations: **un** uncus **t** tegumen **f** lateral fold of uncus **g** gnathos **v** vinculum **sc** saccus **dg** digitiform projection of valve **bv** base of valve **ab** aedeagus base **fp** flattened processes **lc** left curves. Scale bar 0.5 mm.

**Figures 48–55.**
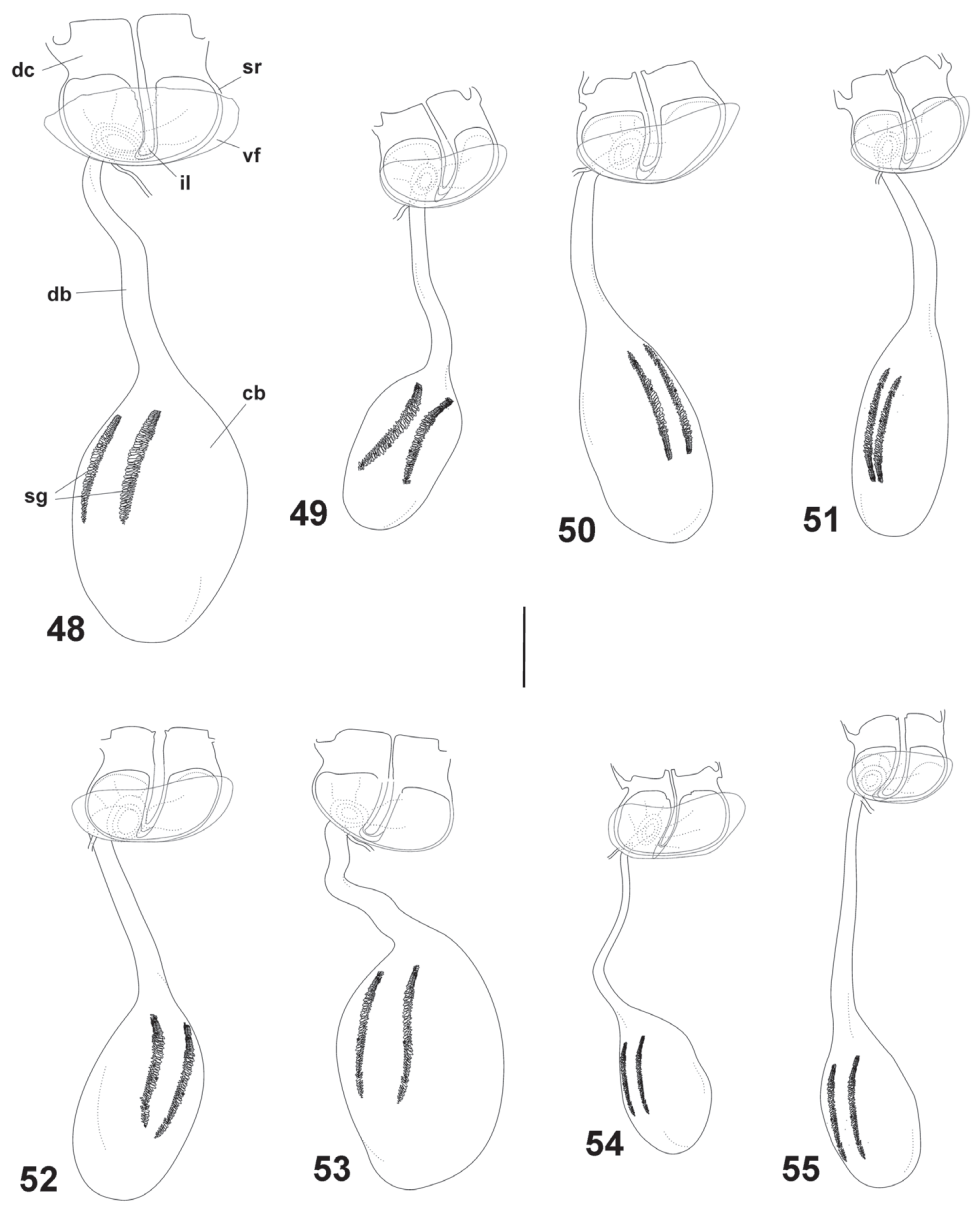
Female genitalia of Cuban *Calisto*, ventral view **48** *Calisto israeli* **49** *Calisto smintheus* **50** *Calisto brochei* **51** *Calisto bruneri* **52** *Calisto muripetens* **53** *Calisto occulta*, new species **54** *Calisto bradleyi* **55** *Calisto herophile*. Abbreviations: **dc** dorsal crown **st** sterigmal ring **vf** ventral fold of sterigmal ring **il** inner loop of sterigmal ring **db** ductus bursae **cb** corpus bursae **sg** signa. Scale bar 0.5 mm.

**Figures 56–59.**
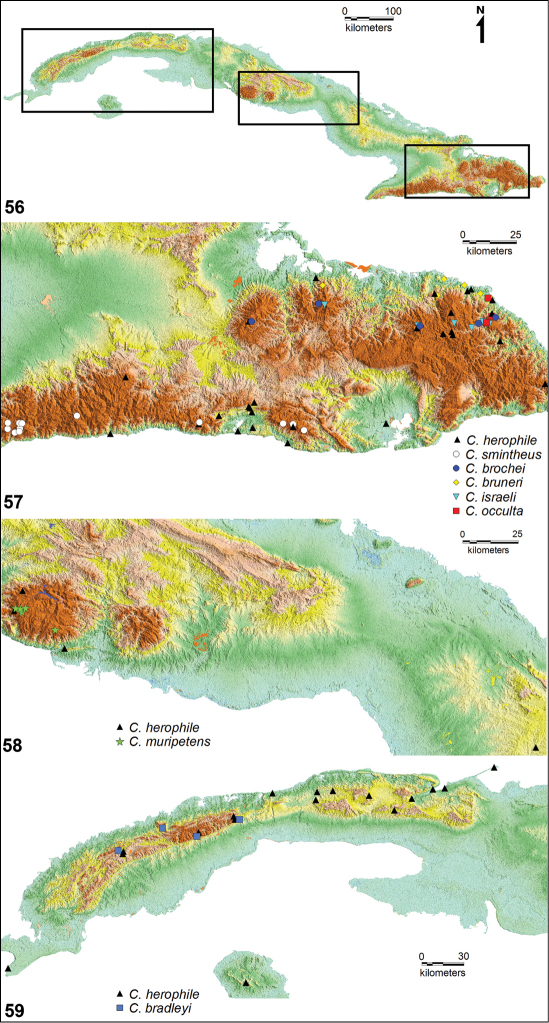
Geographical distribution of Cuban species of *Calisto*. **56** Location of four Cuban major mountains ranges **57** Right rectangle in figure 56, distribution of *Calisto israeli*, *Calisto brochei*, *Calisto bruneri*, *Calisto occulta*, new species, and*Calisto herophile* at Nipe– Sagua– Baraoca mountain range, north, and *Calisto smintheus* and*Calisto herophile* at Sierra Maestra range, south**58** Central rectangle in figure 56, distribution of *Calisto muripetens* and *Calisto herophile* at Guamuhaya mountain range, central Cuba **59** Left rectangle in figure 56, distribution of *Calisto bradleyi* and *Calisto herophile* at Guaniguanico mountain range, western Cuba.

**Figures 60–65.**
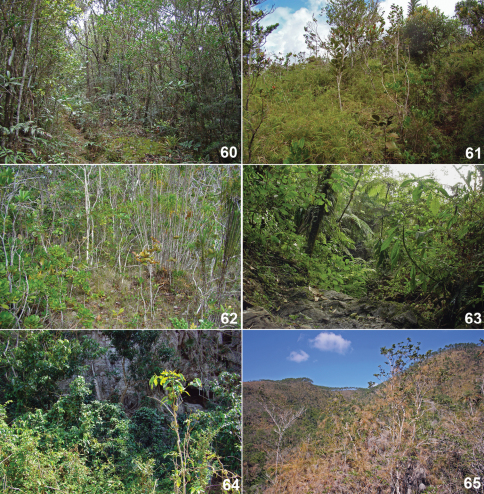
Habitat of Cuban *Calisto*. **60** Rainforest path at north slope of Monte Iberia plateau, 600 m, habitat of *Calisto israeli*, *Calisto brochei*, and *Calisto occulta*, new species **61** Path dominated by climbing grasses, *Arthrostylidium pinifolia* and *Chusquea* sp., at north slope of Monte Iberia plateau, 400 m, preferred situation by *Calisto israeli*, *Calisto occulta*, new species, is also present but not abundant **62** Scrub forest (charrascal) at Yamanigüey, habitat of *Calisto bruneri*, *Calisto occulta*, new species, and in lesser degree of *Calisto israeli* **63** Lower strata of rainforest at Aguada de Joaquín, Sierra Maestra, habitat of *Calisto smintheus* **64** Secondary forest at base of limestone hill, mogote, at Viñales valley, habitat of *Calisto bradleyi* **65** Dry scrub on serpentine soil at Cajálbana, habitat of *Calisto bradleyi*.

**Figure 66.**
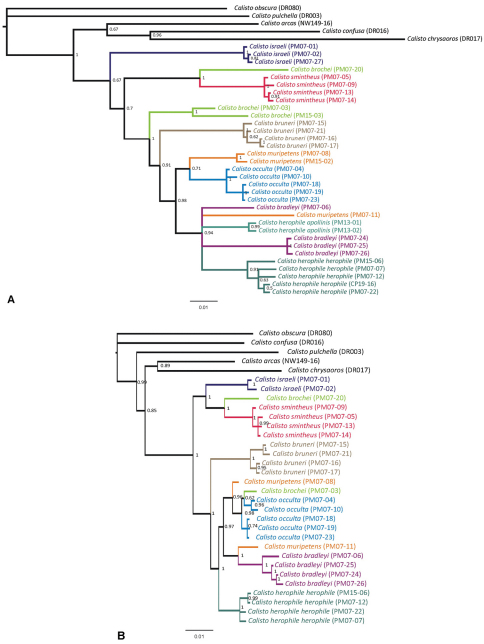
**A** Phylogenetic hypothesis based on a Bayesian analysis of COI data **B** Phylogenetic hypothsis for Cuban *Calisto* based on five nuclear gene regions. For both figures, numbers to the right of nodes give the posterior probability of the node. Lineages leading to species are coloured.

### Calisto smintheus

Bates, 1935 stat. rev.

http://species-id.net/wiki/Calisto_smintheus

Figs 4–6 26 33 41 49 56, 57 63 66 –74

Calisto smintheus Bates 1935: 242
Calisto delos Bates 1935: 243, Michener 1943: 6, Schwartz and Hedges 1991: 136
Calisto smintheus smintheus Bates 1939: 3, Michener 1943: 6, Munroe 1950: 226, Torre 1952: 62, Torre 1954: 120, Torre 1968: 18, Núñez 2009: 56
Calisto smintheus delos Torre 1968: 19
Calisto biocellatus Torre 1968: 22
Calisto sibylla smintheus Brown and Heineman 1972: 51, Fontenla and Rodríguez 1990: 8, Smith et al. 1994: 57, Lamas 2004: 207
Calisto sibylla delos Brown and Heineman 1972: 51, Smith et al. 1994: 57, Lamas 2004: 207

#### Diagnosis.

*Calisto smintheus* requires comparison with some of its cogeners. Within Cuba, the more similar species is *Calisto brochei*,but *Calisto smintheus* adults are larger on the average (19–25 mm of FWL versus 16–22 mm in *Calisto brochei*), have a reddish suffusion around anal lobe at the UPHW, and are darker and more brightly colored at UN of wings. The androconial patch has a rounded outer margin in *Calisto smintheus*, but it is sinuous, forming three rounded lobes in *Calisto brochei*.Almost all other Cuban relatives except *Calisto israeli*, are paler and have fewer white dots at the post discal area on UNHW. *Calisto herophile* has also four white dots at UNHW, but is paler and smaller on the average, 14–21 mm versus 19–25 mm in *Calisto smintheus*. Outside Cuba, the Bahamian *Calisto sibylla* lacks red at the UNFW cell and the reddish suffusion at anal lobe; and in general, is a paler species. The Hispaniolan *Calisto confusa* Lathy, 1899, *Calisto hysius* (Godart [1824]) and *Calisto obscura* Michener, 1943, and *Calisto pauli* Johnson & Hedges, 1998 are superficially similar but all are distinctly smaller (13–18 mm) than *Calisto smintheus*.

#### Description.

FWL: 19–25 mm ♂ & ♀. Male UPFW dark brown except darker, almost black, androconial patch and postdiscal area adjacent to androconial patch and tornus, pale brown (Fig. 4). Androconial patch distinct except at base anterior limit, approximately triangular with outer margin rounded, anterior margin not entering into cell, about one half the length of FW (Fig. 33). Female UPFW dark grayish brown at basal two thirds, outer third pale grayish brown (Fig. 5). UPHW dark grayish brown at anterior two thirds, pale grayish brown at posterior third; anal lobe ferruginous, occupying apical half of posterior margin in some specimens. UN of wings brown heavily mixed with reddish and, toward base, pale yellow scales; apex of both wings and basal to pdl of **HW** with a dark wine hue (Figs 6, 26). Outer edge of pdl with bright yellow scaling. Post discal area at UNHW with four white dots at Rs–M_1_, M_1_–M_2_, M_2_–M_3_, and M3–Cu_1_, the last one slightly displaced toward outer margin and smaller, sometimes absent in rubbed specimens. Male genitalia with tegumen about two thirds the length of uncus, rounded at posterior half (Fig. 41); uncus gradually tapering toward apex, arched at apical third; digitiform projection of valvae slender and long, straight at both margins; aedeagus sinuated with a left curve both at basal and apical half. Female genitalia with dorsal crown tall (Fig. 49); corpus bursae broad, about two thirds the length of ductus bursae.

**Figures 67–74.**
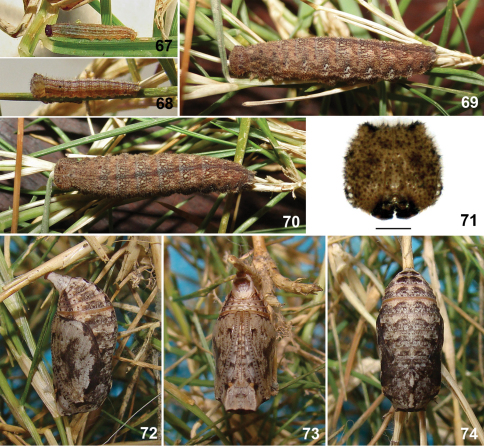
Immature stages of *Calisto smintheus*. **67** First instar **68** Second instar **69** Fifth instar, lateral view **70** Fifth instar, dorsal view **71** Fifth instar head capsule, scale bar 1 mm. **72** Pupa, lateral view **73** Pupa, ventral view **74** Pupa, dorsal view.

#### Type material.

Holotype♂: Sierra del Cobre, Loma del Gato 3000 ft, 20°00'33"N, 76°02'16"W, 25–30/IX/1935, S. C. Bruner. MCZ, examined. Paratypes 8 ♂, 4 ♀: same data as for holotype except 2700–3300 ft, S. C. Bruner, genitalia ♂ & ♀ in glycerin. MCZ, CZACC, examined.

*Calisto delos* Bates 1935: holotype ♂, Ote (currently Santiago de Cuba), Pico Turquino, Loma Cordero (actually Cardero) 4000–6000 ft, 1 August 1935, J. Acuña; paratype ♂, Pico Turquino, Julio 22 de 1922, S. C. Bruner & C. H. Ballou, EEA Cuba No. 1652. MCZ, examined.

*Calisto biocellatus* Torre 1968: holotype ♂, Turquino, Pico Cuba 1872 m, 19°59'8.4"N, 76°50'32.3"W, VI/1963, F. de Zayas, P. Alayo & I. García; allotype ♀: same data as for holotype. CZACC, examined.

#### Additional material.

88 ♂, 33 ♀. **Granma:** Bartolomé Masó, La Platica 850 m, 20°00'54.1"N, 76°53'28.4"W, 26/XI/2007, R. Núñez, slide RNA175(androconial sclaes), DNA voucher PM07–05 (3 ♂); same data as for anterior except V/2008 (2 ♂). **Santiago de Cuba:** Aguada de Joaquín 1300 m, 20°00'50.4"N, 76°50'24.8"W, 20–27/I/2005, A. García, A. Barro & R. Núñez, genitalia ♂ in glycerin, slides RNA238(wings)/243(legs & labial palpus) (2 ♂, 1 ♀); same data as for anterior except 30/XI/2007, R. Núñez, genitalia ♀ in glycerin, slide RNA190(wings) (2 ♂, 1 ♀); Sierra Maetra, Pico Joaquín 5300 ft, 19°59'16"N, 76°53'31"W, 18/V/1948, J. Ferrás (3 ♂); ladera sur Pico Regino 1500 m, 20°00'38"N, 76°50'9"W, 29/XI/2007, R. Núñez, genitalia ♀ in glycerin, DNA voucher PM07–09 (M010) (1 ♂, 1 ♀); Sierra Maestra, 29/X/1941, J. Acuña (1 ♂); Turquino, June 1963, P. Alayo, slide RNA208(wings) (5 ♂); same data as for anterior except F. de Zayas, P. Alayo & I. García (1 ♀); Pico Turquino 1972 m, 19°59'23.7"N, 76°50'11.9"W, 18/X/1966, I. García, slide RNA275(legs & labial palpus) (10 ♂, 4 ♀); same data as for anterior except XII/1967, slides RNA225 (wings)/227(legs & labial palpus) (1 ♂, 1 ♀); same locality as for anterior, X/1985, M. G. Casanova, genitália ♀ in glycerin (1 ♂, 2 ♀); Ote (currently Santiago de Cuba), Turquino, Pico Cuba 1872 m, 19°59'8.4"N, 76°50'32.3"W, VI/1963, F. de Zayas, P. Alayo & I. García, genitalia ♂ & ♀ in glycerin, slides RNA186(androconial scales)/189/204/212(wings)/203/230/266 (legs & labial palpus) (10 ♂, 1 ♀); same locality as for anterior, 17/I/2002, A. Barro & R. Núñez (1 ♀); Ote (currently Santiago de Cuba), Sierra del Cobre, Loma El Gato 2600 ft, 20°00'33"N, 76°02'16"W, 24–30 September 1935, J. Acuña, S. C. Bruner & L. C. Scaramuzza (1 ♂, 1 ♀); same locality as for anterior, VIII/1942, Hno Crisogono (2 ♂); same locality as for anterior, 6/IX/1951, S. L. de la Torre, slide RNA228(wings) (8 ♂, 3 ♀); same locality as for anterior, 17–20 June 1952, F. de Zayas & P. Alayo (3 ♂); same locality as for anterior, 19 June 1952 (3 ♂); same locality as for anterior, 20 June 1952, slide RNA273(legs & labial palpus) (1 ♂); same locality as for anterior, 11/VIII/2008, E. Oliva, genitalia in glycerin, DNA vouchers PM07–13 (M030) & PM07–14 (M031) (2 ♂); same locality and date as for anterior, E. Fonseca (1 ♂); Ote (currently Santiago de Cuba), Caney, Gran Piedra 1100 m, 20°00'31"N, 75°37'3"W, Junio 1954, F. de Zayas & P. Alayo (1 ♀); same locality as for anterior, 23/IV/1955, P. Alayo, genitalia ♂ & ♀ in glycerin (2 ♂, 2 ♀); Ote (currently Santiago de Cuba), Caney, Gran Piedra, El Olimpo 900 m, 20°00'41"N, 75°39'42"W, 22 Mayo 1955, F. de Zayas & P. Alayo, slide RNA234(wings) (1 ♀); same data as for anterior except 26 Abril 1956, genitalia ♂ in glycerin, slides RNA192/221(wings) (4 ♂, 2 ♀); same data as for anterior except VIII/1960, genitalia ♀ in glycerin, slides RNA185(androconial scales)/188/219/251(wings)/216/276(legs & labial palpus) (8 ♂, 3 ♀); same locality as for anterior, VI/1962, P. Alayo, F. de Zayas & I. García (1 ♂); same locality as for anterior, 19/XII/1965 (4 ♂, 1 ♀); same locality as for anterior, 6/X/1966, I. García, genitalia ♀ in glycerin, slide RNA274(legs & labial palpus) (3 ♂, 3 ♀); same locality as for anterior, VIII/1986 (1 ♀); Gran Piedra, base Gran Piedra 1200 m, 16/III/2008, R. Núñez (4 ♂); Gran Piedra, pinar detrás Estación BIOECO 1100 m, 24/II/2011, R. Núñez (1 ♀); same data as anterior except ex ova, emerged 17/V/2011 (1 ♀). MFP, CZACC.

#### Distribution.

Species is restricted to the Sierra Maestra. It has been recorded from Pico Mogote (Fontenla 2006) in the east to 140 km west at La Platica (Figs 56, 57). Besides anterior literature data, species has been recorded from La Bayamesa, Granma province (Fontenla 2005).

#### Immature stages.

 Egg & oviposition – Eggs are glued to substrate, spherical in shape and ivory white in color becoming beige with irregular orange brown spots a day after laid. Time to hatch 8 days (n=1).

First instar larva (Fig. 67) – Head capsule dark brown, almost black, with a bronze gloss and with two short horns on top. Body beige, greenish white on sides after fed on host leaves, with a dorsal line and four pairs of longitudinal pale orange brown lines: subdorsal, suprastigmatal, stigmatal, and infrastigmatal. Suprastigmatal line more greenish and the thinnest one, remainder lines more brownish and broader but subdorsal thinner than stigmatal and infrastigmatal lines. Dimensions (n=1): head capsule width 0.61 mm, head capsule height 0.64 mm, initial total length 2.9 mm, final total length 4.2 mm. Duration (n=1): 15 days.

Second instar with beige brown head capsule with slightly darker marks, body pattern similar to first but with a pair of dots, one at each subdorsal line, at metathorax that is present in remainder instars (Fig. 68). Instars from third and fourth with the same pattern of fifth, described below, but paler, with lines less contrasting, subdorsal and suprastigmatal lines straighter and the stigmatal and infragstigmatal lines distinct.

Fifth instar larva (Figs 69–71) – Head capsule beige regularly speckled with numerous dark brown dots; horns reduced; sides with two pairs of dark brown spots, each pair almost equidistant between them and to dorsal and ventral edges; mandibles black; X– mark of epicranium obsolete, represented only by a small rounded spot at apex of each arm, slightly darker than background. Body pale brown with brown striations; dorsum of each segment with darker “butterfly” like mark formed by small brown striations; lines slightly darker than background, except subdorsal which is pale yellow, lines becoming diffuse toward thorax; each abdominal segment with a transverse ashy gray band at beginning from dorsum to near suprastigmatal line and edged anteriorly by a brown dot at each end; dorsal line edged at beginning of each abdominal segment by two pale yellowish beige dots; a dark brown dot above subdorsal line on metathorax; subdorsal lines thinner than dorsal line, wavy, closer to dorsal line at middle of each segment, ending on caudal tails; suprastigmatal lines wavy following the wave pattern of subdorsal ones with dark brown dot above it near mid way to subdorsal, above it on each segment one pair of diffuse brown dots, one central, larger, and other near posterior margin; stigmatal and infrastigmatal lines diffuse mixed; area behind and below whitish, the latter crossed the infrastigmatal line. Dimensions (n=1): head capsule width 2.55 mm, head capsule height 2.58 mm, initial total length 14 mm, final total length 22 mm. Duration (n=1): 19 days.

Pupa (Figs 72–74) – Head and wing sheaths pale gray; antennae and leg sheaths with regular discontinuous pattern of dark brown dots; a pair of ventral black dots on eyes and another at sides of appendages near abdomen; wing sheaths edged at dorsum by an irregular dark brown large spot at middle; dorsum of thorax and abdomen pale gray with diffuse dark brown striations heavier at sides of dorsal ax forming a large spot on each side; abdomen with a dark brown line on sides, abdomen with a transverse ridge with a pair of more prominent crests on dorsum of segments 1 to 6; last abdominal segment long, stout, cremaster area enlarged, broad. Two days before emergence the dark brown extends covering almost entire thorax, extending gradually until occupying entire surface before emergence. Dimensions (n=1): total length 11 mm, maximum width 4.5 mm. Duration (n=1): 12 days.

#### Habitat and biology.

 Throughout its range, the species inhabits evergreen and rainforests at altitudes between 800 m and 1500 m (Fig. 63). It is also found in cloud forest above 1500 m, and at the cloud scrub around Pico Turquino, 1972 m and Cuba highest peak. Individuals can be found in interior of forests but also at its edges. The species seems to prefer relatively well preserved areas but occasionally can be found at places with secondary vegetation. At La Platica village, Turquino massif, Sierra Maestra, the species was observed in shady places of gardens nearby forest, whereas, at Gran Piedra, it was found inside 25 year old pine plantations. Adults were observed feeding on flowers of *Bourreria laevis*, *Palicourea alpina*, *Pavonia fruticosa*, *Mikania micrantha*, and *Stachyterpheta cayenensis* in rainforest near La Platica.

Two females were observed when laid eggs singly at underside of leaves near midday. The host, *Ichnanthus mayarensis*, is the first one recorded for the Cuban species of the genus.This small grass is common at forest understory, sometimes abundant along paths, of rainforests in the Turquino Massif. Larvae eat the entire corion after hatching and feed at night remaining inactive during the day in lower parts of the plant. Larvae accepted both substitute host plants. First instar was 15 days long and all other were 9 days long each. Prepupal period was one day long and pupal stage extended for 12 days. Immature development takes 80 days and five larval instars.

#### Remarks.

*Calisto smintheus* and *Calisto herophile* are the only members of the genusinhabiting the Sierra Maestra. Their altitudinal ranges overlap between 800 and 1100 m, however, *Calisto herophile* is rare in places where *Calisto smintheus* is present and vice versa. Munroe (1950) mentioned the possibility of hybridization between them but there is no evidence available from present work to confirm it. The phylogenetic inferences and genetic distances agree on the establishment of *Calisto smintheus* as a single species with a minimum divergence of no lower than 5% from other Cuban *Calisto* taxa. The close phylogenetic relationship between *Calisto smintheus* and *Calisto brochei* is discussed below.

### Calisto brochei

Torre, 1973 stat. n.

http://species-id.net/wiki/Calisto_brochei

Figs 7–9 27 34 42 56, 57 60 66 75–82

Calisto smintheus brochei Torre 1973: 6, Núñez 2009: 56
Calisto sibylla smintheus Fontenla and Rodríguez 1990: 8, Lamas 2004: 207

#### Diagnosis.

*Calisto brochei* is similar to *Calisto smintheus* but is smaller on average, lacks the reddish suffusion at the anal lobe in the UPWH, and is paler and less brightly colored at UN of wings (see more details below *Calisto smintheus*). *Calisto brochei* has four white dots on UNHW and the androconial patch trilobed at the outer margin whereas *Calisto bradleyi*, *Calisto occulta*, sp. n., and *Calisto muripetens* have only three white dots and have different shaped androconial patches, the first species with a single rounded lobe at apex, and the other two without lobes at the outer margin. *Calisto herophile* also resembles *Calisto brochei*, but it is paler and has a smaller androconial patch without lobes at the outer margin. From *Calisto sibylla*, *Calisto brochei* differs by its darker coloration, the presence of red in cell at the UNFW, and the three lobes at the outer margin of androconial patch. The Hispaniolan *Calisto confusa*, *Calisto hysius* and *Calisto obscura* are superficially similar but are smaller on the average (13–17.5 mm of FWL),and have straighter white edged lines at UNHW. *Calisto pauli* possesses a similar wing pattern but its female genitalia has a terminal production a middle of dorsal crown, absent in *Calisto brochei*, and its male genitalia has the uncus and tegumen flattened, they are slightly rounded in *Calisto brochei*. Also, the uncus is shorter in *Calisto brochei* and the aedeagus has two prongs at apex, there are four in *Calisto pauli*.

#### Description.

FWL: 16–22 mm ♂, 20–22 mm ♀. Male UPFW dark brown except darker, almost black, androconial patch, outer third slightly paler (Fig. 7). Androconial patch distinct, dark brown almost black, approximately triangular with outer margin waved forming three usually distinct lobes, anterior margin not entering into cell, about one half the length of FW (Fig. 34). Female UPFW dark brown at basal two thirds, outer third pale brown (Fig. 8). UN background brown moderately mixed with pale reddish and pale yellow scaling basal to pdl and apex of both wings (Figs 9, 2^7^). Outer edge of pdl with pale yellow scaling. Post discal area at UNHW with four white dots at Rs–M_1_, M_1_–M_2_, M_2_–M_3_, and M3–Cu_1_, the last one smaller, sometimes absent in rubbed specimens. Male genitalia with tegumen about two fifths the length of uncus, rounded at posterior half (Fig. 42); uncus gradually tapering and arched toward apex, base subquadrated; digitiform projection of valvae heavy and moderately long, ventral margin concave; aedeagus straight at basal half and with a left curve at apical half. Female genitalia with dorsal crown tall (Fig. 50); corpus bursae broad, about the same length of ductus bursae.

**Figures 75–82.**
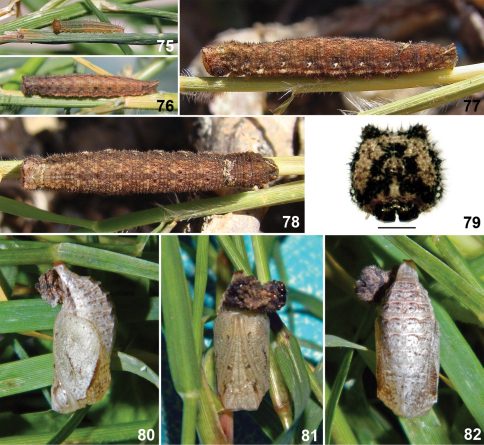
Immature stages of *Calisto brochei*. 75 First instar **76** Fourth instar **77** Sixth instar, lateral view **78** Sixth instar, dorsal view **79** Sixth instar head capsule, scale bar 1 mm. **80** Pupa, lateral view **81** Pupa, ventral view **82** Pupa, dorsal view.

#### Type material.

Holotype♀: Oriente (currently Guantánamo), Cupeyal 730 m, 20°26'57"N, 75°03'38"W, VI/1971, I. García. CZACC, examined. Paratypes 1 ♂, 5 ♀: same data as for holotype, genitalia ♀ in slide. CZACC, MFP, examined.

**Additional material:** 12 ♂, 6 ♀. **Holguín:** Ote (currently Holguín), Pinares de Mayarí 800 m, 20°28'8"N, 75°48'52"W, 16/X/1966, I. García, slide RNA269(wings) (1 ♀); Mayarí, camino de La Zoilita 250 m, 20°38'N, 75°29'W, IX/1986, R. Rodríguez, genitalia ♀ in glycerin (1 ♂, 1 ♀); Sierra de Cristal, cerca de la Estación La Zoilita 400 m, 20°37'41.7"N, 75°29'08.1"W, 15–20/II/2010, R. Núñez, DNA voucher PM07–20 (M037) (1 ♂). **Guantánamo:** same data as for holotype, genitalia ♀ in glycerin, slides RNA224/246/257/261(wings)/277(legs & labial palpus) (3 ♂, 2 ♀); Baracoa, Monte Iberia, campamento ladera norte 600 m, 20°29'25.5"N, 74°43'51.3"W, 18/V/2007, R. Núñez, slide RNA169(wings), DNA voucher PM07–03 (M003) (2 ♂); same data as for anterior except 2/V/2011, ex ova, emerged 9/VIII/2011 (1 ♂, imperfect); Baracoa, Monte Iberia, al sur de las Tetas de Julia 430 m, 20°27'58.6"N, 74°46'9.2"W, 20/V/2007, R. Núñez, slides RNA249(wings)/250(legs & labial palpus), DNA voucher PM15–03 (M049) (1 ♂); Baracoa, Monte Iberia, ladera norte 385 m, 20°29'53"N, 74°43'48"W, 1/V/2011, R. Núñez (3 ♂, 1 ♀). CZACC.

#### Distribution.

*Calisto brochei* is present in several localities in the middle and western NSB mountains, from Monte Iberia plateau to more than 100 km west at Pinares de Mayarí at Nipe plateau (Figs 56, 57). The species is probably present along NSB wherever its habitat is preserved.

#### Immature stages.

 Egg & oviposition – Eggs are glued to substrate. Color is pale yellow with slight greenish tint becoming beige with irregular orange brown spots a day after being laid. Eggs are near spherical, diameter 1.0–1.1 mm, height 0.8–1.0 mm (n=9). Time to hatch 7 to 8 days (n=9).

First instar larva (Fig. 75) – Head capsule pale orange beige, with two short horns on top. Body beige, pale grayish green after fed on host leaves, with a dorsal line and three pairs of longitudinal pale brown lines: subdorsal, suprastigmatal, and stigmatal. Dorsal, subdorsal and stigmatal lines thinner than suprastigmatal one; suprastigmatal and stigmatal lines are closer between them than remainder lines. Dimensions (n=9): head capsule width 0.65–0.68 mm, head capsule height 0.67–0.71 mm, initial total length 2.7–3.0 mm, final total length 4.2–4.5 mm. Duration (n=9): 11–16 days.

Instars from second to fifth (Fig. 76) with color pattern similar to that of sixth, described below, but with pattern better defined and the following dots: a pair, brown, at end of each abdominal segment, the upper one in contact with the more ventral part of subdorsal lines waves; a dark brown, larger at middle segments, at each abdominal segment on the most dorsal portion of suprastigmatal lines waves, above spiracles.

Sixth instar larva (Figs 77–79) – Head capsule beige regularly speckled with scarce dark brown dots; horns reduced, spotted with dark brown; sides with a dark brown vertical line passing horns to epicranial suture; a dark brown band crossing lower part of frons and curved down at sides to stemmata; sides of clypeus, mandibles and stemmatal areas dark brown, almost balck; X– mark of epicranium dark brown, arms ellipse like and connected by almost indistinct paler lines, lower arms larger. Body pale brown with brown striations; dorsum of each segment with darker diffuse X– marks at sides of dorsal line; lines slightly darker than background, diffuse; a transverse diffuse band at end of each abdominal segment, slightly darker than background; dorsal line edged at beginning of each abdominal segment by two dark brown dots; subdorsal lines wavy, diffuse almost indistinct, closer to dorsal line at middle of each segment; suprastigmatal lines wavy following the wave pattern of subdorsal ones; stigmatal and infrastigmatal lines diffuse, indistinct, area between them and below paler; spiracles dark gray brown surrounded by whitish. Dimensions (n=1): head capsule width 2.62 mm, head capsule height 2.69 mm, initial total length 18.4 mm, final total length 22 mm. Duration (n=1): 15 days.

Pupa (Figs 80–82) – Entirely pale ashy gray minutely speckled with darker gray color heavier dorsolaterally on wing sheats; three pairs of frontal brownish gray dots: one elongated on eyes and two smaller and rounded on sheaths of legs, one at first third and the other at apical third; wing sheaths with a small darker crescent on the middle; a row of small submarginal dots on wing sheats; abdomen with a transverse ridge with a pair of more prominent crests on dorsum of segments 1 to 6, with a brownish gray line on sides; last abdominal segment long, stout, cremaster area large, broad. Two days before emergence eyes turns dark brown extending gradually to occupying entire surface. Dimensions (n=1): total length 10 mm, maximum width 4.3 mm. Duration (n=1): 12 days.

#### Habitat and biology.

 The species inhabits several variants of rain and evergreen forests of NSB Mountains at altitudes between 200 and 800 m (Fig. 60). Individuals can be found mainly at shady forest paths.

Larvae eat the entire corion after hatching and feed at night, remaining in the lower parts of grasses during day. *Calisto brochei* larvae did not accept well the two grass species supplied as substitute food and only one of nine larvae survived to pupation. Average duration of each instar was about two weeks each. Prepupal period was two days long and pupal stage extended for 12 days. Immature development took three months and larvae went through six instars (possibly due to low food quality). Adult emergence occurred at the beginning of the afternoon, between 14:00 and 15:00.

#### Remarks.

 Although superficially almost identical, *Calisto brochei* and *Calisto smintheus* both possess small consistent differences in the adult stage and are well differentiated in the immature stages. Adults can be separated by the characters given above in the Diagnosis section. Immature stages are more different than adults, with the first instar of *Calisto smintheus* having the head capsule dark brown, almost black, whereas it is pale orange in *Calisto brochei*. In the latter species, the pair of dark dots on dorsum of metathoraxpresent from secondinstar of *Calisto smintheus* is absent. Pupae are also different, with those of *Calisto brochei* paler in color pattern whereas in *Calisto smintheus* have a heavily dark spotted pattern.

Two individuals of *Calisto brochei* (PM07-03 and PM15-03) are grouped together by the COI sequences (Fig. 66A) and placed as sister to a large clade comprising all other taxa except *Calisto israeli* and *Calisto smintheus*. One of these individuals (PM07-03) was also sequenced for nuclear genes, which place it within the new species *Calisto occulta* (Fig. 66B). Another individual (PM07-20) is the sister to *Calisto smintheus* based on both sets of markers, but is morphologically different to *Calisto smintheus*. The existence of hybrids between *brochei* and *occulta* may explain these results. Munroe (1950) hypothesized that populations of *Calisto smintheus* and *Calisto herophile* may interbreed. The high mortality rate of *Calisto brochei* larvae may be due to substitute food used during rearing; however, it could be also an indication of hybridization. It is clear that more individuals of *Calisto brochei* need to be sequenced in order to discover the general patterns of molecular variation in this species.

### Calisto bruneri

Michener, 1949 stat. rev.

http://species-id.net/wiki/Calisto_bruneri

Figs 10–12 28 35 43 51 56, 57 60–62 66

Calisto bruneri Michener 1949: 2, Torre 1952: 62, Torre 1954: 120, Torre 1968: 17, Núñez 2009: 56
Calisto herophile bruneri Alayo and Hernández 1987: 40, Smith et al. 1994: 56, Lamas 2004: 207

#### Diagnosis.

*Calisto bruneri* differs from all other *Calisto* with similar color pattern, brown with red at the UNFW cell, by its pear shaped ocellus at the UNHW, ovoid in the others. *Calisto bruneri* was regarded in the past as subspecies of *Calisto herophile*; however, besides the character given above, the first has uniformly colored UP of wings and three white dots at UNHW, whereas the second has the apical third of wings paler and four white dots. Their genitalia also differ, that of *Calisto bruneri* male has valvae with concave ventral margins and a more sinuous aedeagus in dorsal view, and its female genitalia has the ductus and corpus bursae almost equal in length, the ductus is almost twice the length of the corpus in *Calisto herophile*. The Hispaniolan *Calisto pulchella* Lathy, 1899, *Calisto raburni* Gali, 1985, *Calisto schwartzi* Gali, 1985 and *Calisto tasajera* González, Schwartz & Wetherbee, 1991 also have pear shaped ocelli but can be easily separated from *Calisto bruneri*, among other features, by the conspicuous reddish suffusion at the UN of wings that is absent in the latter.

#### Description.

FWL: 16–19 mm ♂, 18–21 mm ♀. UP of wings uniform dark grayish brown almost black, anal lobe with a small black dot (Figs 10, 11). Androconial patch indistinct in fresh specimens, approximately triangular with apex slightly angled, anterior margin not surpassing posterior margin of cell, about two fifths the length of FW (Fig. 35). UNHW background brown mixed with grayish white and, in less extent, pale yellow scales (Figs 12, 28). UNHW ocellus pear shaped and narrow. Post discal area on UNHW with three white dots at M_1_–M_2_, M_2_–M_3_, M_3_–Cu_1_, the first one smaller and sometimes absent in rubbed specimens. Male genitalia with tegumen about two thirds the length of uncus, slightly curved (Fig. 43); uncus broad at basal third, tapering gradually from the middle toward apex, arched at apical third, base with a small ventral notch; valvae base very broad; digitiform projection of valvae narrow and short with ventral margin concave; aedeagus sinuated in dorsal view with a left curve both at basal and apical half, the first one more pronounced. Female genitalia with dorsal crown tall (Fig. 51); corpus bursae somewhat narrow, near equal in length to ductus bursae.

#### Type material.

Holotype♂: Oriente (currently Holguín), Moa, 20°39'23"N, 74°56'34"W, 24–27 February 1948, F. de Zayas & J. Ferrás. AMNH, not examined. Paratypes 6 ♂, 1 ♀: same data as for holotype (5 ♂, 1 ♀); same locality as for holotype 13–22 April 1945, J. Acuña (1 ♂). MCZ, CFZ, examined.

#### Additional material.

13 ♂, 9 ♀. **Holguín:** same data as for holotype: (1 ♀); Moa, El Johnson 300 m, 20°35'36.4"N, 74°59'9.9"W, Junio 1954, F. de Zayas & P. Alayo, genitalia in glycerin (1 ♀); same data as for anterior except 5/I/1968, S. L. de la Torre, genitalia ♀ in glycerin, slides RNA161/211/214(wings)/183/215/217(legs & labial palpus) (4 ♂, 3 ♀); Oriente (currently Holguín), Moa, Punta Gorda o Cayo del Medio, 20°37'44"N, 74°51'10"W, 6/I/1968, S. L. de la Torre, slide RNA255(legs & labial palpus) (1 ♀); Moa, Cayo Grande, 20°35'28.9"N, 74°46'52.6"W, 19/I/2009, R. Núñez & E. Oliva, genitalia in glycerin, slides RNA256(legs)/267(FW) (1 ♂); same data as for anterior except 24/I/2009, genitalia ♂ & ♀ in glycerin, slides RNA252/258(legs)/253 (wings)/254(legs & labial palpus), DNA vouchers PM07–15 (M032) & PM07–16 (M033) (4 ♂, 2 ♀); Moa, Yamanigüey 75 m, 20°34'45.9"N, 74°44'10.2"W, 24–27/IX/2009, R. Núñez, DNA voucher PM07–17 (M034) (2 ♂, 1 ♀); Sierra de Cristal, cerca de Estación La Zoilita 400 m, 20°37'41.7"N, 75°29'08.1"W, 15–20/II/2010, R. Núñez, DNA voucher PM07–21 (M038) (2 ♂). CZACC, MFP.

#### Distribution.

*Calisto bruneri* occurs in the western parts of the NSB Mountains (Figs 56, 57). Previous records gave a small distribution area around Moa town, including Punta Gorda, Holguín province, near the coast up to an altitude of 300 m in neighboring hills (Michener 1949; Torre 1968). Its distribution is widened here about 10 km east to Cayo Grande, and 55 km westward to Sierra de Cristal at 450 m of altitude.

#### Immature stages.

Torre (1968) mentioned that females glued eggs to the substrate and that they are spherical and beige with orangish spots.

#### Habitat and biology.

The species inhabits rainforests, scrub forests (charrascales) and pine forests (Figs 60–62). Scrub forests have high levels of sun exposition and water loss, and *Calisto bruneri* has been observed to spend most time near the ground in the shade of shrubs. At Cayo Grande, Moa, the species was observed taking nectar from flowersof *Scaevola wrightii*, a local endemic shrub. Throughout its range, the species is replaced by *Calisto herophile* in areas where its habitat has been destroyed, mostly around towns and major roads.

#### Remarks.

Alayo and Hernández (1987) considered *bruneri* a subspecies of *Calisto herophile* arguing that UNHW ocellus shape was the only difference.Like previous authors (Michener 1949; Torre 1968), Núñez (2009) considered it a valid species. Morphological and molecular support of its species status are discussed below.

DNA analysis of *Calisto bruneri* (4 specimens, 3 localities) showed an average divergence of 4.68% from *Calisto herophile* (5 specimens, 5 localities). Indeed *Calisto bruneri* forms a single well-supported monophyletic clade together with *Calisto occulta*, *Calisto muripetens*, *Calisto bradleyi* and *Calisto herophile*, and altogether sister to the group *israeli-brochei-smintheus* (Fig. 66). Furthermore, both nuclear and mitochondrial datasets suggest an earlier divergence of *Calisto bruneri* from sister taxa within the clade, diversifying later in the western part of the NSB Massif. Average COI distances between *Calisto bruneri* and *Calisto occulta*, *Calisto muripetens* and *Calisto bradleyi* are 4.94%, 5.52% and 5.52% respectively.

### Calisto muripetens

Bates, 1939 stat. n.

http://species-id.net/wiki/Calisto_muripetens

Figs 13–15 36 44 52 56, 58 66

Calisto smintheus muripetens Bates 1939: 3, Michener 1943: 6, Munroe 1950: 226, Torre 1952: 62, Torre 1954: 120, Torre 1968: 20
Calisto sibylla muripetens Fontenla and Rodríguez 1990: 8, Smith et al. 1994: 57, Lamas 2004: 207

#### Diagnosis.

*Calisto muripentens* is similar to several Cuban congeners. From the more similar *Calisto bradleyi* and *Calisto occulta*, both with three white dots at the UNHW with the middle one distinctly larger, *Calisto muripetens* differs by its androconial patch, without the apical lobe present in the first and occupying a larger area of wing than in the second. Their female genitalia are also different, being the corpus bursae smaller in *Calisto muripetens* than in *Calisto occulta*, and the dorsal crown taller in the first than in *Calisto bradleyi*.It differs from *Calisto smintheus*, *Calisto brochei*,and *Calisto herophile*, which have four white dots at the UNHW, by having only three white dots at that part of wings with the one at M_2_–M_3_ interspace distinctly larger. Other differences with *Calisto smintheus* and *Calisto brochei* are detailed in their respective Diagnosis sections. From *Calisto herophile*, it also differs by the larger area occupied by its androconial patch and its size, larger on the average, 18–22 mm of FWL versus 14–19 mm in males, and 20–23 mm versus 17–21 mm in females. The Hispaniolan *Calisto confusa*, *Calisto hysius*, *Calisto obscura*,and *Calisto pauli* are superficially similar but are smaller,and have four white dots at the UNHW.

#### Description.

FWL: 18–22 mm ♂, 20–23 mm ♀. Male UPFW uniform grayish brown except androconial patch, dark brown almost black (Fig. 13). Androconial patch distinct from surrounding areas, about one half the length of FW, approximately triangular in shape with apex and outer margin rounded, anterior margin entering into cell (Fig. 36). Male UPHW dark grayish brown, paler at outer third. Female UP of wings uniform grayish brown, paler than male (Fig. 14). UNFW cell red patch variable in size, occupying from apical third to entire cell. Pdl edged by scarce pale yellow scaling. HW background brown mixed with pale yellow and, in less extent, ochre scales (Fig. 15). Post discal area on UNHW with three white dots at M_1_–M_2_, M_2_–M_3_, M3–Cu_1_, with that on M_2_–M_3_ larger, smaller dots can gone in rubbed specimens. Male genitalia with tegumen about two thirds the length of uncus, dorsally flat and posteriorly rounded (Fig. 44); uncus gradually tapering and curved from base to apex, base rounded; valvae base broad; digitiform projection of valvae short and stout with ventral margin slightly concave; aedeagus straight at basal two thirds with a left curve at apical third in dorsal view. Female genitalia with dorsal crown tall (Fig. 52); corpus bursae somewhat broad, near equal in length to ductus bursae.

#### Type material.

Holotype♂: Trinidad Mountains, Buenos Aires 2500–3500 ft, 21°59'13"N, 80°11'20"W, 8–14 May 1936, P. J. Darlington. MCZ, not examined. Paratypes 1 ♂, 2 ♀: same locality as for holotype, 4 May 1932, S. C. Bruner & A. Otero. MCZ, not examined.

#### Additional material.

 11 ♂, 4 ♀. **Cienfuegos:** same locality as for holotype, 16/VI/1967, slide RNA272(wings) (1 ♀); carretera a Pico San Juan, V/1986, J. L. Fontenla, slide RNA268(wings)/284 (legs & labial palpus) (3 ♂); Pico San Juan 1140 m, 21°59'25"N, 80°08'50"W, V/2006, R. Núñez, DNA voucher PM15–02 (M048) (3 ♂); Carso de Buenos Aires 725 m, 21°59'13"N, 80°11'20"W, V/2006, R. Núñez, genitalia ♀ in glycerin, slides RNA197/236(wings), DNA vouchers PM07–08 (M009), PM07–11 (M018) (1 ♂, 1 ♀); ladera norte de Pico Cuevita 900 m, 21°59'13"N, 80°10'18"W, V/2006, R. Núñez, genitalia ♂ in glycerin, slides RNA193(androconial scales)/200(legs & labial palpus)/235 (wings) (1 ♂). **Sancti Spiritus**: Topes de Collantes, Mi Retiro 800 m, 21°53'41"N, 80°01'02"W, V/2002, R. Núñez, genitalia ♂ & ♀ in glycerin, slides RNA166/199/241(wings) /209/210(legs & labial palpus), DNA voucher PM15–01 (M047) (3♂, 2♀). CZACC.

#### Distribution.

*Calisto muripetens* is restricted to a few localities in the central Cuban mountains: the Guamuhaya massif, above 750 m and up to 1140 m on Pico San Juan, the highest peak (Figs 56, 58).

#### Immature stages.

 Unknown.

#### Habitat and biology.

The species inhabits evergreen forests of the mogotes vegetation complex, limestone hills of vertical slopes, and rainforests, flying mostly in shady places.

#### Remarks.

*Calisto smintheus muripetens* type series was not available for study. Online pictures of MCZ insect type material, last accessed in 9^th^ October 2011, do not include them. However, examination of original description leaves no doubt of its identity. *Calisto muripetens* differs from *Calisto herophile*, the only other species in its range, by its larger size, darker color pattern and structure of the genitalia of both sexes.

*Calisto muripetens* is closest to *Calisto occulta*, a new species described below from NSB, the northeastern Cuban mountain range. Besides differences noted at the Diagnosis section, *Calisto muripetens* has other differences with *Calisto occulta*. These include the proportionally larger genitalia of the latter with the aedeagus with an enlarged base, swollen both in dorsal and lateral view.

As with *Calisto brochei*, two individuals of *Calisto muripetens* (PM07-08 and PM07-11) did not group together in both the nuclear and the mitochondrial data analyses (Fig. 66). A third individual, PM15-02, groups together with PM07-08 in the COI tree in a clade sister to *Calisto occulta*. The relationships of PM07-11 are unresolved in the mitochondrial data set being located in an unresolved clade containing *Calisto herophile* *s.l.* and *Calisto bradleyi*; however, this individual is sister to *Calisto bradleyi* based on the nuclear markers (Fig. 66B). This pattern suggests either hybridization or retained ancestral polymorphisms (see Discussion for further discussion on the potential causes of polyphyletic multiple haplotypes in *Calisto*).

### Calisto occulta

Núñez sp. n.

urn:lsid:zoobank.org:act:96685BEF-1929-4005-802D-F5C3C82BD2C4

http://species-id.net/wiki/Calisto_occulta

Figs 16–18 29 37 45 53 56, 57 60–62 66 83–89

Calisto sp., Núñez 2009: 56

#### Diagnosis.

*Calisto occulta* is more similar to *Calisto muripetens* and *Calisto bradleyi* than other Cuban relatives. Characters separating *Calisto occulta* from *Calisto muripetens* are discussed above, at the Diagnosis section of the latter. From *Calisto bradleyi*, *Calisto occulta* differs by its darker color, its androconial patch without apical lobe, the slight red suffusion below cell at the UNFW, and its proportionally larger male and female genitalia. From the remainder Cuban species and from Bahaman ones with similar pattern, *Calisto occulta* can be separated by having fewer white dots at the UNHW (except *Calisto bruneri*), its proportionally larger male and female genitalia, and the presence of a slight red suffusion below the cell at UNFW.The Hispaniolan *Calisto confusa*, *Calisto hysius*, *Calisto obscura*,and *Calisto pauli* are superficially similar but are smaller,and have four white dots at the UNHW.

#### Adult.

 Male (Figs 16, 18, 29, 37) – FWL: 17–20 mm. *Head*: antennae dark brown, UN pale yellow at basal third and UP orange at club; eyes black, hairy, delimited by a pale yellow band; labial palpi dark brown on UN, pale yellow on UP, middle and basal segments rough. *Thorax*: UPFW uniform grayish brown except androconial patch, dark brown almost black. Androconial patch slightly distinct from surrounding areas, about two fifths the length of FW, approximately triangular in shape with apex rounded, anterior margin entering into cell and apex reaching M_3_ origin (Fig. 37). UPHW darker than FW, about the same hue of androconial patch. UNFW brown, slightly posterior to pdl (Figs 18, 29); a red patch in outer half of cell with outer margin edged by dl, patch posterior margin diagonal between anterior and posterior limits of cell; a slight red scaling below cell; dl, pdl and both stl darker than background; basal third of costa and outer edge of pdl with grayish white scales; ocellus black encircled by a pale yellow ring laying M_1_–M_3_, with two white pupils laying midway between M_1_– M_2_ and M_2_–M_3_, the posterior one more basad. UNHW brown mixed with pale yellow and grayish white scales; pdl and stl outer edged with pale yellow scales around ocellus; pdl area with three white dots at M_1_– M_2_, M_2_–M_3_ and M_3_–Cu_1_, with that on the middle greatly enlarged, dots surrounded by scattered whitish lilac scales; ocellus large, broad, laying between Cu_1_ and Cu_2_, black with a bluish white pupil at base and surrounded by a yellowish ochre ring outer edged by a ferruginous suffusion; tornal lobe slightly developed, black, innerly edge with pale yellow; legs dark brown, inner side of femora pale yellow, tibiae and tarsi white on external side. *Abdomen*: UP dark brown, UN pale yellowish brown. *Genitalia* (Fig. 45): uncus having typical bird’s beak shape with a dorsal keel and gradually tapering toward apex, arched at apical half, base protuberant and rounded, separated from tegumen by a single dorsal notch; tegumen hood shaped, dorsally flat but rounded at anterior end, approximately one half the length of uncus, lateral fold narrow, extending ventrally along vinculum; gnathos spine shaped, approximately 0.3 the length of uncus; valvae elongated with a broad base, digitiform projection of valvae stout with a very broad base and slightly concave at venter, extending toward apical third of uncus, joins to main body relatively sclerotized; saccus developed, finger–like at anterior half and flattened, slightly convex, toward venter on posterior half; aedeagus robust and slightly arched ventrad at middle, straight at basal two thirds with a strong left curve at apical third in dorsal view, moderately swollen toward basal half both in lateral and dorsal view, ventrally divided from basal third to bifid terminus, ending in a pair of ventral triangular flattened processes.

Female (Fig. 17) – FWL: 18–21 mm. Similar to male except: UP of wings uniform dark brown; UNFW with red scaling below cell more distinct than in male, below lower limit of cell. *Genitalia* (Fig. 53): large in proportion to body; sterigmal ring rounded and well developed, dorsal crown broad and symmetrical, ring almost entirely covered by a ventral fold slightly sclerotized; inner sterigmal loop large, sclerotized, left arched in ventral view almost reaching anterior margin of ring; ductus bursae originated at left side of sterigmal ring in ventral view, membranous; ductus seminalis arising close to origin of ductus bursae; corpus bursae greatly enlarged, broad, approximately the same length of ductus bursae, signa formed by two parallel columns of numerous transverse rows of small irregular sclerotized processes.

**Figures 83–89.**
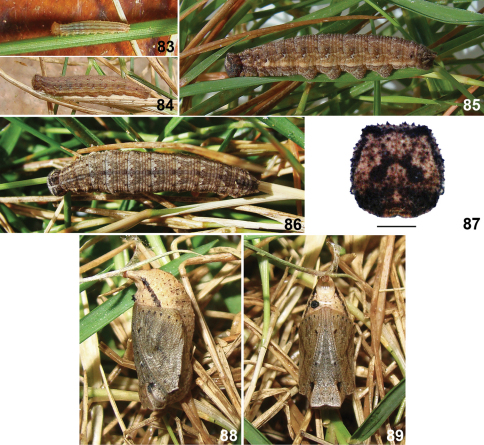
Imatures stages of *Calisto occulta*, new species. **83** First instar **84** Fourth instar **85** Sixth instar, lateral view **86** Sixth instar, dorsal view **87** Sixth instar head capsule, scale bar 1 mm. **88** Pupa, lateral view **89** Pupa, ventral view.

#### Holotype.

♂: Guantánamo, Baracoa, Monte Iberia plateau, Tetas de Julia 650 m, 20°27'47"N, 74°45'13.3"W, 20/V/2007, R. Núñez, DNA voucher PM07–10 (M017). CZACC.

#### Paratypes.

 7 ♂, 3 ♀: Holguín, Moa, Yamanigüey 75 m, 20°34'46.5"N, 74°45'12.2"W, 24–27/IX/2009, R. Núñez, DNA voucher PM07–23 (M041) (1 ♂, 1 ♀); same data as for anterior except: ex ova, emerged 28/I/2010, DNA voucher PM07–18 (M035) (1 ♂); same data as for anterior except: emerged 31/I/2010, DNA voucher PM07–19 (M036) (1 ♀); same data as for holotype except genitalia ♂ & ♀ in glycerin, DNA voucher PM07–04 (M004) (1 ♂, 1 ♀); Monte Iberia plateau, campamento ladera norte 600 m, 20°29'25.5"N, 74°43'51.3"W, 18/V/2007, R. Núñez, genitalia in glycerin, slide RNA165(wings) (1 ♂); Baracoa, Monte Iberia, ladera norte 385 m, 20°29'53"N, 74°43'48"W, 1/V/2011, R. Núñez (3 ♂). CZACC.

#### Etymology.

 The species name derives from the Latin *occultus* (hidden, reserved) in reference to the cryptic nature of this species that remained hidden between its sympatric congeners until the present work.

#### Distribution.

*Calisto occulta* is known from a few localities from the middle part of the NSB Mountains, from the Monte Iberia plateau 14 km north to near Yamanigüey, in northeastern Cuba (Figs 56, 57). It is probable that *Calisto occulta* is more widespread in the NSB in areas where its habitat is preserved.

#### Immature stages.

 Egg & oviposition – Eggs are glued to substrate, are spherical in shape and ivory white in color becoming beige with irregular orange brown spots a day after being laid. Time to hatch 8 to 9 days (n=7).

First instar larva (Fig. 83) – Head capsule pale orange beige, with two short horns on top. Body beige, bluish white after fed on host leaves, with a dorsal line and three pairs of longitudinal pale brown lines: subdorsal, suprastigmatal, and stigmatal. Dimensions (n=7): head capsule width 0.60–0.62 mm, head capsule height 0.63–0.66 mm, initial total length 2.6–2.7 mm, final total length 3.5–3.8 mm. Duration (n=7): 13–15 days.

Second to fifth instars (Fig. 84) with the color pattern similar to that of sixth, described below, but paler and less contrasting and without the tranversal ashy gray bands.

Sixth instar larva (Figs 85–87) – Head capsule beige brown with numerous dark brown dots, a vertical dark brown line from each side reaching horns and joining at epicranial suture, a dark brown line connecting horns with subdorsal lines, horns much reduced; ventral third dark brown, almost balck, with a small rounded pale beige area at frons near clypeus; mandibles amber brown; X–mark of epicranium black with lower arms longer and rounded at tip. Body pale grayish brown, yellow from above spiracles to above prolegs, ventral side, including prolegs brown; dorsum of each segment with a darker “butterfly” like mark formed by small brown striations; lines slightly darker than background, except subdorsal which is pale yellow; each abdominal segment with a transverse ashy gray band at beginning from dorsum to suprastigmatal line; dorsal line edged at beginning of each segment by two black dots encircled in ashy gray; subdorsal lines thinner than dorsal line, wavy, closer to dorsal line at posterior margin of each segment, ending on caudal tails, with black dots on its upper edge aligned with dots of dorsal and suprastigmatal lines; suprastigmatal lines thin, diffuse, above it on each segment a central white dot encircled in brown and another, brown, near posterior margin; stigmatal lines thinner passing dorsal to spiracles encircled in ashy gray; infrastigmatal line thin and diffuse. Dimensions (n=2): head capsule width 2.41–2.57 mm, head capsule height 2.53–2.68 mm, initial total length 14–16 mm, final total length 22–23 mm. Duration (n=2): 30–35 days.

Pupa (Figs 88–89) – Head and wing sheaths pale brown with a row of black dots at wing sheaths margin; three pairs of frontal black dots: one elongated on eyes and two smaller and rounded on sheaths of legs, one at first third and other nearer to apex; wing sheaths edged on thorax by a brown line; dorsum of thorax and abdomen pale yellow with transverse rows of tiny black dots, density varies between individuals giving a darker or paler appearance to abdomen; abdomen smooth, with a brown line on sides; last abdominal segment long, stout, cremaster area reduced. Three days before emergence color turns brown on dorsum extending gradually to occupying entire surface. Dimensions (n=2): total length 11–12 mm, maximum width 4.5–4.7 mm. Duration (n=2): 18–19 days.

#### Habitat and biology.

The species inhabits the scrub forests (charrascales) of lowlands and rainforests up to 700 m in the NSB mountain range (Figs 60–62). At Yamanigüey scrub, it flies mostly below shrub shadow avoiding the high temperatures of insolated areas.

Larvae eat the entire corion after hatching and feed at night, remaining in the lower parts of grasses during day. *Calisto occulta* larvae did not accept well the two grass species supplied as substitute food and only two of seven larvae survived to pupation after undergoing six instars. Duration of first four instars was about two weeks each whereas fifth and sixth took about three and five respectively. Prepupal period was two to three days long and pupal stage extended for two and a half weeks. Immature development took up to four months. Adult emergence occurred at the beginning of the afternoon, between 14:00 and 15:00. A mated pair was observed at 3:00 pm Monte Iberia north slope in May 2011.

#### Remarks.

It isremarkable that the closest species to *Calisto occulta* is *Calisto muripetens*, an inhabitant of another mountain range. The relationship between them was discussed above. In the following paragraphs we discuss the differences with the remainder Cuban taxa.

Immature stages also support species status. The first instar of *Calisto occulta* has a pale orange beige head capsule which is almost black in *Calisto smintheus* and *Calisto herophile*. The longitudinal lines are fewer more spaced on sides and dorsum in *Calisto smintheus* and *Calisto occulta* than in *Calisto herophile*. Larvae of fifth and sixth instars of *Calisto occulta* have transverse ashy gray bands at beginning of each segment occupying from dorsum to suprastigmatal line, those lines are absent from *Calisto herophile* larvae. The subdorsal brown dots at metathorax of *Calisto smintheus* are absent in *Calisto occulta*.Pupae also show differences. Those of *Calisto herophile* have several pair of ridges on dorsum of abdomen and are beige, almost immaculate. In *Calisto occulta*, the head and thorax are pale grayish brown and the abdomen, that lacks the dorsal ridges, is beige with numerous black dots and a dark brown stripe at sides. As whole, is more spotted than the pupa of *Calisto herophile* but less than *Calisto smintheus*. Pupal head and cremaster shape are also different between species. Development time and number of larval instars also differ. The complete development took 60 to 70 days in *Calisto herophile* and 80 in *Calisto smintheus* both with five instars and 99 to 120 days and six larval instars in *Calisto occulta*.

The DNA analyses place all *Calisto occulta* (5 specimens, 2 localities) together, although the nuclear data placed a specimen of *Calisto brochei* within the *Calisto occulta* clade (Fig. 66). Both datasets suggest that *Calisto occulta* is related to *Calisto muripetens*, *Calisto bradleyi* and *Calisto herophile*, perhaps with *Calisto muripetens* being the closest relative. The species is separated from *Calisto herophile* and *Calisto bradleyi* with an average COI distance of 2.28% and 3.09% respectively, while the average COI divergence within *Calisto occulta* sampled from two distinct localities is just 0.98%.

### Calisto bradleyi

Munroe, 1950 comb. n.

http://species-id.net/wiki/Calisto_bradleyi

Figs 19–21 30 38 46 54 56 59 64–66

Calisto smintheus bradleyi Munroe 1950: 227, Torre 1952: 63, Torre 1954: 121, Torre 1968: 7
Calisto sibylla bradleyi Brown and Heineman 1972: 51, Alayo and Hernández 1987: 41, Fontenla and Rodríguez 1990: 9, Smith et al. 1994: 57, Lamas 2004: 207

#### Diagnosis.

*Calisto bradleyi* resembles *Calisto muripetens* and *Calisto occulta* more than its other congeners. It can be separated from these species by the presence of an apical lobe at the androconial patch, and by having an iridescent blue band edging the black dot of the anal lobe at the UNHW. Other differences were treated in the Diagnosis section of those species. From other Cuban (except *Calisto bruneri*), Hispaniolan and Bahamian species differs by the same characters and by have fewer white dots at UNHW. Its female genitalia is also diagnostic due to its proportionally smaller size and its thinner dorsal crown. The Hispaniolan *Calisto confusa*, *Calisto hysius*, *Calisto obscura*, and *Calisto pauli* are superficially similar but are smaller,and have four white dots at the UNHW.

#### Description.

FWL: 17–20 mm ♂, 20–21 mm ♀. UPFW outer third and area anterior to apical half of androconial patch pale grayish brown, basal area anterior to patch darker (Figs 19, 20); costal two thirds and androconial patch dark brown, almost black. UPHW uniform dark brown, slightly paler than androconial. Androconial patch distinct from surrounding areas, approximately triangular with a rounded lobe at apex, not entering into cell, about one half the length of FW (Fig. 38). Lines at UN of wings with little if any pale shade of external side (Figs 21, 30). UNHW background pale brown heavily mixed with ochre scaling basal to pdl. Post discal area on UNHW with three white dots at M_1_– M_2_, M_2_–M_3_, M3–Cu_1_, with that on M_2_–M_3_ larger, smaller dots can gone in rubbed specimens. UNHW lobe with a black dot anteriorly edged by a small band of iridescent blue scales. Male genitalia with tegumen about half the length of uncus, tapering gradually toward apex and arched along its length (Fig. 46); uncus strongly arched; digitiform projection of valvae stout, slightly arched toward venter; aedeagus with two sinuations of left side at apical half, the basal one smaller. Female genitalia small (Fig. 54); dorsal crown of sterigmal ring very narrow; corpus bursae small and broad, about two fifths the length of ductus bursae; ductus bursae very thin.

**T**

#### ype material.

Holotype♂: Pinar del Río, Sierra de Rangel (currently Sierra del Rosario), Río Tacoluco (almost surely Río Taco Taco), 3 March 1939, J. C. Bradley. Location unknown, not examined.

#### Additional material.

 14 ♂, 13 ♀. **Pinar del Río:** Viñales 150 m, X/1985, J. L. Fontenla, genitalia ♂ & ♀ in glycerin, slides RNA202(legs & labial palpus)/260(wings) (1 ♂, 2 ♀); no collection data but probably the same same as for anterior, genitalia ♂ & ♀ in glycerin, slides RNA181 (androconial scales) /244/245/262/263/270/271 (wings)/247/ 265/278(legs & labial palpus) (6 ♂, 7 ♀); cuabales ladera sur de Cajálbana 150 m, 22°46'33.1"N, 83°26'22.1"W, III/2002, R. Núñez, DNA voucher PM15–08 (M054), genitalia in glycerin (1 ♀); Viñales, base norte mogote Dos Hermanas 140 m, 22°37'16.4"N, 83°44'40.3"W, 17/IV/2009, R. Núñez & E. Oliva, DNA vouchers PM07–24 (M043), PM07–25 (M044) & PM07–26 (M045) (6 ♂, 3 ♀). **Artemisa**: Pinar del Río (currently Artemisa), Sierra del Rosario, El Taburete 300 m, 22°50'11"N, 82°55'24"W, 9/X/2007, R. Núñez, DNA voucher PM07–06 (M006), genitalia in glycerin (1 ♂).

#### Distribution.

*Calisto bradleyi* occurs in the major mountain range of western Cuba, Guaniguanico, from El Taburete, at Sierra del Rosario, 90 km west to Viñales valley, always at low elevations (Figs 56, 59). The species was previously known only from the type locality, Rangel, and Viñales (Munroe 1950; Fontenla 1987b). Attempts to find it at the type locality were made by Torre (1968) and ourselves without success. Here we recorded it for the first time from Cajálbana and El Taburete widening its distribution to the eastern most portion of Guaniguanico mountain range.

#### Immature stages.

 Unknown.

#### Habitat and biology.

The species inhabits various vegetation types throughout its distribution but can only be found in areas where original elements are still dominant. Habitats include the evergreen forest at El Taburete, the mogote vegetation complex at Viñales, and the dry scrub on serpentine soil at Cajálbana (Figs 64, 65). In Viñales valley, Pinar del Río, the species was flying in the shadow of the base of mogotes (limestone hills of almost vertivcal slopes) appearing occasionally in sunny places. There it was observed feeding on flowersof *Stachyterpheta cayenensis*, *Hyptis verticilla*, and *Urena lobata*, and a mating pair was observed at 3:30 pm in April 2009.

#### Remarks.

The type specimen of *Calisto smintheus bradleyi* is apparently lost. Searching of the type specimen at the different collections mentioned by Munroe (1950), CUIC, AMNH, MCZ, and CMNH, was fruitless. However, based on the examination of original description and since the other only species in its range of distribution, *Calisto herophile*, is rather different, it can be easily identified.

DNA analyses are somewhat ambiguous about the relationships of *Calisto bradleyi*. The mitochondrial dataset suggests that *Calisto bradleyi* is paraphyletic with regard to *Calisto herophile* and one individual of *Calisto muripetens* (Fig. 66), while the nuclear data place the monophyletic *Calisto bradleyi* in a clade with *Calisto occulta* and *Calisto muripetens*. The COI distance between the sister species *Calisto herophile* and *Calisto bradleyi* is 1.91%. Nonetheless, the status of species in both cases is still valid as the molecular phylogenies consistently separate the lineages (Fig. 66). Therefore, we prefer to treat them as separate entities, proposing the species status for *Calisto bradleyi*, potentially phylogenetically close to *Calisto herophile*.

### Calisto herophile

Hübner, 1823

http://species-id.net/wiki/Calisto_herophile

Figs 22–24 31 39 47 55 56–59 66 90 –99

Calisto herophile Hübner 1823: 16, Gundlach 1881: 111, Lathy 1899: 223, Dethier 1940: 14
Satyrus herophile Poey, 1847: 179
Calisto herophile herophile Bates 1935: 242, Michener 1943: 6, Michener 1949: 1, Munroe 1950: 225, Torre 1952: 62, Torre 1954: 120, Torre 1968: 12, Brown and Heineman 1972: 51, Alayo and Hernández 1987: 39, Schwartz and Hedges 1991: 136, Smith et al. 1994: 56, Lamas 2004: 2007, Núñez 2009: 56

#### Diagnosis.

*Calisto herophile* can be separated by its similar congeners in several ways. From *Calisto smintheus* and *Calisto brochei*,it differs, among other features, by its paler background color at both sides of wings, the inconspicuousness of its androconial patch and its less sclerotized male genitalia with a shorter uncus and less sinuous aedeagus. From *Calisto muripetens*, *Calisto occulta* and *Calisto bradleyi*,it differs by having four white dots and paler coloration. Differences with *Calisto bruneri* are detailed in the Diagnosis section of that species. It is also similar the Bahamian *Calisto sibylla* but smaller, 14–21 mm of FWL versus 23 mm in *Calisto sibylla* which also lacks the red in cell at the UNFW present in *Calisto herophile*. The Hispaniolan *Calisto confusa*, *Calisto hysius* and *Calisto obscura* although similar in size are darker,and have straighter white edged lines at the UNHW. Other Hispaniolan species, *Calisto pauli*, is similar in size and pattern but has different genitalia including a larger and flattened uncus in males and a terminal production in the dorsal crown of the female genitalia.

#### Description.

FWL: 14–19 mm ♂, 17–21 mm ♀. Male UP of wings dark brown at basal area more or less defined by UN pdl, area outer to pdl distinctly paler (Fig. 22). Androconial patch indistinct in fresh specimens, approximately triangular with apex slightly angled, anterior margin not surpassing posterior margin of cell, about two fifths the length of FW (Fig. 39). Female UP of wings as in male but distinctly paler (Fig. 23). UNHW background pale brown heavily mixed with pale yellow scales (Figs 24, 31). Post discal area on UNHW with four two white dots at Rs–M_1_, M_1_–M_2_, M_2_–M_3_, M_3_–Cu_1_ interspaces, the last one, and occasionally the first one too, smaller and sometimes absent in rubbed specimens. Male genitalia with tegumen about two thirds the length of uncus, nearly straight, posterior end rounded (Fig. 47); uncus broad at basal half, tapering gradually from the middle toward apex, arched at apical third; digitiform projection of valvae with ventral margin straight; aedeagus only slightly sinuated in dorsal view, with two small left curves at apical half. Female genitalia with dorsal crown tall (Fig. 55); corpus bursae somewhat broad, about 0.6 the length of ductus bursae.

**Figures 90–97.**
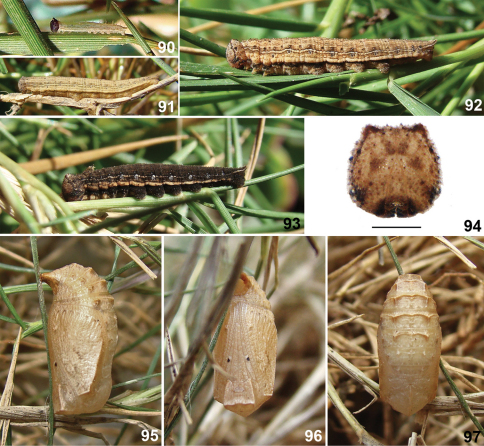
Immature stages of *Calisto h. herophile*. **90** First instar **91** Fourth instar **92** Fifth instar, pale morph **93** Fifth instar, dark morph **94** Fifth instar head capsule, scale bar 1 mm. **95** Pupa, lateral view **96** Pupa, ventral view **97** Pupa, dorsal view.

**Figures 98–99.**
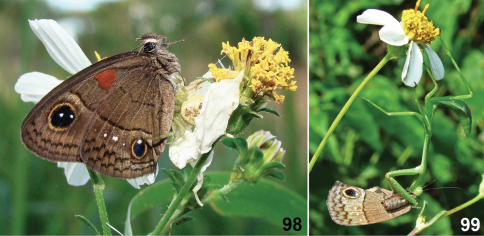
Predation on *Calisto h. herophile* **98** Predation by a crab spider, Thomisidae, November 2008 at La Chata, La Habana province **99** Predation by a mantis nymph, *Stagmomantis domingensis*, July 2009 at Pan de Matanzas, Matanzas province.

#### Type material.

Holotype♂: Cuba, Havannah. Location unknown, not examined.

#### Additional material.

 148 ♂, 76 ♀. **Pinar del Río:** Pinares de Viñales 200 m, 22°35'N, 82°42'41"W, V/1963, P. Alayo & I. García, genitalia in glycerin, slide RNA 223(legs & labial palpus) (1 ♂); Rangel 400 m, 22°45'N, 83°11'W, 2/XI/1966, I. García & S. L. de la Torre (4 ♂); same data as for anterior except I. García, slide RNA196(wings) (1 ♀); same locality and collector as for anterior 21/VII/1967 (9 ♂, 6 ♀); same locality as for anterior, R. Núñez & E. Oliva, 19–20/IV/2009, ex ova, emerged 19/VI/2009 (2 ♂); same data as for anterior except emerged 20/VI/2009 (1 ♀); 22/VI/2009 (1 ♂); 23/VI/2009 (1 ♀); 26/VI/2009 (1 ♂); Viñales 150 m, 22°36'59"N, 82°42'28"W, 21/VII/1967 (6 ♂); same locality as for anterior, X/1985, J. L. Fontenla (2 ♂); Valle de Viñales, 9/I/1974, A. Castiñeiras (1 ♂); carretera a Viñales km 22 200 m, 22°34'29"N, 82°42'11"W, 14/I/1974, A. Castiñeiras (1 ♀). **Mayabeque:** Jaruco, Cueva Don Martin, 23°00'N, 82°01'W, 4/V/1966 (5 ♂); La Habana (currently Artemisa), Guajaibón próximo a Mariel, 23°01'N, 82°40'52"W, 25/V/1967 (1 ♂, 1 ♀); same locality as for anterior, X/2007, R. Núñez, DNA voucher PM07–07 (M008) (1 ♀); Pinar del Río (currently Artemisa), Sierra del Rosario, III/1968, R. González (1 ♂); Pinar del Río (currently Artemisa), Sierra del Rosario, alrededores Estación Biológica 180 m, 22°51'N, 82°55'53"W, 1–10/X/2007, R. Núñez (1 ♂); Sierra del Rosario, El Mulo 200 m, 22°51'29"N, 82°56'54"W, 10/X/2007, R. Núñez (1 ♂). **Isla de La Juventud:** Isla de Pinos (currently Isla de La Juventud), Cerro San Pedro 150 m, 21°42'47"N, 82°51'50"W, 20/X/1966, I. García (2 ♂); Habana (currently Isla de La Juventud), Isla de Pinos (currently Isla de La Juventud), 30/X/1966, I. García (7 ♂, 7 ♀). **Habana:** Cerro, 23°06'27"N, 82°23'20"W, 9/I/1934 (1 ♂); Arroyo Naranjo, 23°01'N, 82°22'W, 5 August 1935, L. C. Scaramuzza (1 ♀); Santiago de Las Vegas, 22°58'N, 82°23'W, 15/VIII/1935, S. C. Bruner, genitalia in glycerin (1 ♀); same locality as for anterior, 5 Marzo 1946, J. Ferrás (1 ♂); same data as for anterior except 19 March 1948 (2 ♂); Cotorro, 23°02'N, 82°16'W, 1/XII/1947, J. T. Sierra (1 ♂). **Mayabeque:** Matanzas (currently Mayabeque), 5 km W Ceiba Mocha 150 m, 22°58'50"N, 81°46'24"W, 8/IX/1940, S. L. de la Torre (1 ♂); La Habana (currently Mayabeque), Madruga, La Jiquima 125 m, 22°53'58"N, 81°50'34"W, 5/X/1948, S. L. de la Torre & J. Ortiz (1 ♀). **Matanzas:** Los Prácticos, 23°02'37"N, 81°34'32"W, 23/VII/1940, S. L. de la Torre (1 ♂); Playa, 23°02'37"N, 81°34'32"W, 11/V/1942, S. L. de la Torre (1 ♀); same data as for anterior except 16/VI/1942, slide RNA242(wings) (1 ♂); same data as for anterior except 29/VIII/1947 (1 ♀); same data as for anterior except 6/X/1947 (1 ♂); same data as for anterior except 26/VIII/1948 (1 ♀); same data as for anterior except 27/VIII/1948 (1 ♂); same data as for anterior except 6/XI/1948 (2 ♂); km 6 Vía Blanca, Playa Mamey, 23°03'06"N, 81°29'41"W, 6/VII/1953, S. L. de la Torre (1 ♀); Varadero, Varahicacos, 23°11'40"N, 81°09'16"W, 17/VI/2008, R. Núñez, slide RNA218(wings), DNA voucher PM15–04 (2 ♂). **Cienfuegos:** Las Villas (currently Cienfuegos), Escambray, Mina Carlota 450 m, 22°03'55"N, 80°09'38"W, 15/VI/1967, genitalia ♂ & ♀ in glycerin, slides RNA207(legs & labial palpus)/206(wings) (3 ♂, 2 ♀); Las Villas (currently Cienfuegos), Escambray, Buenos Aires 700 m, 21°59'13"N, 80°11'20"W, 16/VI/1967, genitalia ♂ & ♀ in glycerin, slides RNA182(androconial scales)/203(legs & labial palpus)/226/232(wings) (9 ♂, 4 ♀); Escambray, Charco Hediondo a 10 km de Aguacate, VIII/1978, L. R. Hernández (1 ♂). **Villa Clara:** Mordazo, 22°38'29"N, 80°26'58"W, V/1934 (1 ♀). **Sancti Spiritus:** Trinidad, La Vigía 200 m, 21°48'48"N, 79°58'34"W, 15/VI/1967 (1 ♂). **Camagüey:** Camagüey, 21°22'51"N, 77°55'01"W, 23/IX/1967, S. L. de la Torre (6 ♂). **Holguín:** Ote (currently Holguín), Pinares de Mayarí 800 m, 20°28'8"N, 75°48'52"W, 16/X/1966, I. García (10 ♂, 5 ♀); same locality as for anterior, VI/1967, P. Alayo (1 ♂, 2 ♀); Moa, El Johnson 300 m, 20°35'36.4"N, 74°59'9.9"W, 5/I/1968, S. L. de la Torre, slide RNA 167(wings) (1 ♂); same data as for anterior except 6/I/1968 (1 ♂); Moa, Quemado del Negro, 22°36'40"N, 74°49'22"W, 6/I/1968, S. L. de la Torre (1 ♂, 1 ♀); same data as for anterior except 7/I/1968, slide RNA281(legs & labial palpus) (3 ♂, 3 ♀); Mayarí, camino de La Zoilita 250 m, 20°38'N, 75°29'W, IX/1986, R. Rodríguez, genitalia in glycerin (2 ♂); Mayarí, El Purio, 20°39'45"N, 75°30'55"W, IX/1986, R. Rodríguez, genitalia ♀ in glicerin, slide RNA220(wings) (2 ♂, 2 ♀); Jaguaní, Arroyo Bueno o La Melba 200 m, 20°26'24"N, 74°48'46"W, VIII/2001, R. Núñez (1 ♂, 1 ♀); antiguo campamento minero Meseta de El Toldo 815 m, 20°27'35"N, 74°53'53"W, V/2008, E. Pérez (3 ♂); Moa, km 1 camino de La Melba, 20°36'12"N, 74°50'20"W, 19/I/2009, R. Núñez, genitalia ♀ in glycerin, slide RNA259(legs & labial palpus), DNA voucher PM15–06 (M052) (1 ♂, 2 ♀); Moa, Yamanigüey 75 m, 20°34'45.9"N, 74°44'10.2"W, 24/I/2009, R. Núñez, slide RNA264(wings), DNA voucher PM157–05 (2 ♂, 1 ♀); ♀), Sierra de Cristal, cerca de la Estación La Zoilita 400 m (20°37'41.7"N, 75°29'08.1"W), 15–20/II/2010, R. Núñez, DNA voucher PM07-22 (M040). **Santiago de Cuba:** Las Lagunas, 19°59'37"N, 75°47'50"W, 29/VI/1930 (1 ♂); Santa María, 20°05'N, 75°49'W, Julio 1940, slides RNA 177/178(androconial sclaes) (2 ♂, 1 ♀); same locality as for anterior, 18 May 1941, slide RNA180 (androconial scales) (1 ♂); same locality as for anterior, 20 Junio 1943 (1 ♂); same locality as for anterior, 29 Junio 1943 (1 ♂); Marimón, 27 Junio 1942, slide RNA179(androconial scales) (1 ♂); same locality as for anterior 28 Junio 1942, slide RNA205(wings) (1 ♀); Ote (currently Santiago de Cuba), Ciudamar, 19°58'41"N, 75°51'51"W, 22/IX/1950, S. L. de la Torre (1 ♀); Cuabitas (20°03'48"N, 75°48'05"W, 28/IV/1953, S. L. de la Torre (1 ♂); same locality as for anterior XII/1956, P. Alayo (1 ♀); Las Manuelas camino a Baire 420 m, 20°13'09"N, 76°21'52"W, 23/XI/1952, S. L. de la Torre (1 ♂); Pico Turquino 1972 m, 19°59'23.7"N, 76°50'11.9"W, 18/X/1966, I. García (1 ♀); Ote (currently Santiago de Cuba), Loma El Gato 1000 m, 20°00'33"N, 76°02'16"W, VIII/1942, Hno Crisogono (1 ♂); same locality as for anterior, 6/IX/1951, S. L. de la Torre, genitalia ♂ in glycerin (5 ♂, 5 ♀); same locality as for anterior, 17–20 June 1952, F. de Zayas & P. Alayo (1 ♂); same locality as for anterior, 20 June 1952, slide RNA187(wings)/222(legs & labial palpus) (2 ♂); same locality as for anterior, 25–26 Junio 1952, F. Zayas & P. Alayo (1 ♂); same locality as for anterior, 11/VIII/2008, E. Oliva, DNA voucher PM07–12 (M029) (1 ♂, 1 ♀); same locality and date as for anterior, E. Fonseca (1 ♀); Puerto Boniato, 28/XI/1950, S. L. de la Torre (1 ♂); same data as for anterior except 16/V/1953 (1 ♂); zona del Caney, Loma del Ermitaño 430 m, 20°02'38"N, 75°37'3"W, 13/III/1953 (1 ♂); Ote (currently Santiago de Cuba), Caney, Gran Piedra, 20°00'31"N, 75°42'31"W, Junio 1954, F. de Zayas & P. Alayo, slide RNA229(wings) (2 ♀); same locality as for anterior, 23/IV/1955, genitalia in glycerin (1 ♀); same locality as for anterior, VI/1962, P. Alayo, genitalia in glycerin (1 ♀); Juraguá próximo a Santiago de Cuba, 19°55'31"N, 75°38'28"W, 9/I/1968, S. L. de la Torre (1 ♂); alrededores Estación BIOECO Gran Piedra 1000 m, 20°00'31"N, 75°37'3"W, 16–18/XI/2005, R. Núñez (1 ♀); same data as for anterior except 8/III/2008, genitalia ♀ in glycerin (1 ♂, 1 ♀); same locality as for anterior, 14/VIII/2008, E. Oliva (1 ♂); km 19 carretera Gran Piedra, 12/III/2008, R. Núñez (1 ♂); Gran Piedra, El Olimpo, campamento forestal “Las Marianas", 13/III/2008, R. Núñez, DNA voucher PM15–07 (M053) (2 ♂). **Guantánamo:** Ote (currently Guantánamo), Guantánamo, 20°01'N, 75°12'W, 26/XI/1950, S. L. de la Torre & P. Alayo (1 ♀); Ote (currently Guantánamo), Baracoa, Loma La Farola, 1/V/1968, S. L. de la Torre (2 ♂, 1 ♀); Ote (currently Guantánamo), Cupeyal 730 m, 20°26'57"N, 75°03'38"W, VI/1971, I. García (2 ♂); Piedra La Vela 650 m, 20°24'45"N, 74°56'51"W, VII/2001, R. Núñez (2 ♂); Piedra La Vela, Loma El Mulo 615 m, 20°25'27"N, 74°54'32"W, VII/2001, R. Núñez (1 ♂); río Jaguaní, Vázquez Abajo 560 m, 20°25'15"N, 74°54'33"W, Cuchillas del Toa, Boca de Jaguaní 130 m, 20°22'46"N, 74°41'36"W, VIII/2001, R. Núñez (1 ♂); Yumurí del Sur 450 m, 20°11'21"N, 74°29 31"W, 20/I/2009, R. Núñez & E. Oliva (2 ♂, 2 ♀). CZACC, MFP.

#### Distribution.

 The species is present across the Cuban archipelago from coastal areas to mountains up 1100 m (Figs 56–59).

#### Immature stages.

 Egg & oviposition – Eggs are laid loose, near spherical in shape and ivory white in color becoming beige with irregular orange brown spots a day after laid. Torre (1968) also mentioned that eggs are laid loose. Surface is covered by a fine raised reticulation forming minute polygonal areas (Dethier 1940, Torre 1968). Time to hatch 7 to 9 days (n=16), according Dethier (1940) 6 to 11 and Torre (1968) gave 5 to 8 days.

First instar larva (Fig. 90) – Head capsule dark brown, almost black, with a bronze gloss and with two short horns on top. Body beige, greenish white after fed on host leaves, with a dorsal line and four pairs of longitudinal pale brownish green thin lines all of same width and more or less equally spaced: subdorsal, suprastigmatal, stigmatal and infrastigmatal. Dimensions (n=16): head capsule width 0.52–0.57 mm, head capsule height 0.56–0.59 mm, initial total length 2.2–2.5 mm, final total length 3.4–3.7 mm. Duration (n=16): 7–10 days. This description agrees with that by Dethier (1940), who reported an instar duration of 7 days.

Second to fourth instars (Fig. 91) with the same pattern of fifth, described below, but paler and less contrasting.

Fifth instar larva (Figs 92, 94) – Pale morph. Head capsule pale brownish gray with numerous slightly darker dots, base of setae dark brown, a vertical brown line from each side reaching horns and almost joining at epicranial suture, horns reduced; stemmatal area, clypeus and area around mandibles brown or dark brown; mandibles amber brown, black at edge; X–mark of epicranium slightly darker than background with lower arms longer and rounded at tip, broken as four spots in some specimens. Body pale brownish yellow minutely striated in brownish gray thin lines on dorsum between subdorsal lines, with a dorsal line and five pairs of longitudinal pale brownish gray lines: subdorsal, suprastigmatal, stigmatal and infrastigmatal; dorsal line brownish gray edged at beginning of each segment by two black dots; subdorsal lines somewhat diffuse toward segments margins, with a black dot on its lower edge at posterior margin of each segment, dots on thorax enlarged, lines ending at caudal tails; suprastigmatal lines dark brown, thin, above it on each segment a central white dot encircled in black and another, black near posterior margin; stigmatal lines dark brown, thin, space between it and suprastigmatal pale beige, contrasting, edged on its lower edge by spiracles which are dark and encircled in grayish white; infrastigmatal lines thin, somewhat diffuse; subventral lines thick, wavy, and darkest; ventral side, including prolegs pale brownish yellow. Dimensions (n=4): head capsule width 1.41–1.57 mm, head capsule height 1.55–1.62 mm, initial total length 12–15 mm, final total length 20–23 mm. Duration (n=9): 11–18 days. Larvae reared by the senior author match Dethier (1940) descriptions of instars two to fourth, in general, color pattern is about the same, including the fifth instar, with minor variations.

Dark morph (Fig. 93). Head with all tones darkened. Body background pale brown with lines dark brown, somewhat diffuse; dots at edges of mid dorsal and subdorsal and encirclement of spiracles ashy white, contrasting; a thin pale yellowish beige line between subdorsal and suprastigmatal line, contrasting; dots above suprastigmatal line and encirclement of white dots above it indistinct; space between infrastigmatal and subventral offline pale yellowish beige, contrasting; subventral line thicker than in pale morph, dark brown extending over dorsum of prolegs. Torre (1968) apparently also reared larvae of this morph but only mentioned the general darkening of coloration.

Pupa (Figs 95–97) – Entirely more or less uniform stramineous; one pair of black dots at first third of legs sheaths; abdomen with a transverse ridge with a pair of more prominent crests on dorsum of segments 1 to 6; last abdominal segment short and stout, cremaster area enlarged, broad. Three days before emergence color turns brown on dorsum extending gradually to occupying entire surface. Dimensions (n=9): total length 10–11 mm, maximum width 3.5–4.5 mm. Duration (n=9): 8–10 days.

#### Habitat and biology.

*Calisto herophile* inhabits many habitats, from suburban areas at major cities to the edges of evergreen and rainforests up to 1100 m of altitude, always disturbed in some degree. Individuals can be found any month of the year throughout the island. The species is one of the commonest butterflies in Cuba, especially in altered land with predominantly herbaceous vegetation but shaded to some degree (Fontenla 1987a; Núñez and Barro 2003; Fernández 2007). Fernández (2007) recorded it in Camagüey province from groves, hedges and open scrub land and recorded 26 plant species as nectar sources. We recorded two predation events on this species, one in November 2008 at La Chata, La Habana province, by a crab spider, Thomisidae (Fig. 98); the other in July 2009 at Pan de Matanzas, Matanzas province, by a nymph of the mantid *Stagmomantis domingensis* Palisot de Beauvois (Fig. 99).

Larvae eat the entire corion after hatching and feed at night, remaining in the lower parts of grasses during the day. They accepted well the substitute grasses supplied. Duration of first three instars was about one to one and half weeks each whereas the last two were around two weeks each. The prepupal stage duration was one day long and the pupal stage extended for eight to ten days. Immature development takes 60 to 70 days and goes through five larval instars. Adult emergence occurred after mid day. Dethier (1940) apparently did not complete the life cycle, describing it only to the fourth instar without mentioning the pupa or adult emergence. Dethier used several grass species as food and said that the larvae preferred lawn grass; however, he did not give scientific names of any grass species. Torre (1968), although successful in rearing the species, only described the cycle superficially and mentioning the duration, 70 to 73 days, and number of larval instars, four. He used as substitute food *Saccharum officinarum*, *Zea mays*, and *Stenotaphrum secundatum*, and noted that larvae grew slower with the first.

#### Remarks.

*Calisto herophile* is one of the easiest to recognize among all Cuban *Calisto* species. Its smaller size on average, as well as its pale wing pattern allow their unequivocal identification, although some specimens from altitudes above 800 m can be distinctly larger. The genitalia and immature stages can be also diagnostic. The species has a wide ecological range and tolerance to anthropogenic habitat alteration.

The status of *Calisto herophile* subspecies, *Calisto herophile parsonsi* Clench, 1943and *Calisto herophile apollinis*, is yet pending further investigation. In the present study, only old material of *parsonsi* was available. The unique morphological difference with the nominal subspecies is the more homogeneous pattern at UN of wings, as pointed out by Clench (1943). Genitalic comparisons revealed an identical morphology. We were able to sequence a small fragment (337 bp) of COI for two specimens of the Bahamian subspecies *Calisto herophile apollinis* Bates. These specimens were clearly quite different to Cuban *Calisto herophile* (Fig. 66) and might warrant species status. Future studies involving fresh specimens, immature stages and DNA data could clarify the status of both of these taxa.

### Key to the adults of Cuban Calisto based on wing pattern and geographic distribution

**Table:** 

1	UNFW cell without red spot; UNHW with a large white triangle shaped spot	*Calisto israeli*
–	UNFW cell red spotted, UNHW without a large white triangle shaped spot	2
2	Four white dots on post discal area at UNHW, dot at M_3_–Cu_1 _the smallest and sometimes absent in rubbed specimens	3
–	Less than four white dots on post discal area at UNHW, dot at Rs– M_1 _always absent	5
3	UN of wings background pale brown heavily mixed with pale yellow; male with outer third of UPFW distinctly paler than basal two thirds; androconial patch indistinct	*Calisto herophile*
–	UN of wings background brown mixed with pale yellow, ochre and reddish scales; male with UPFW uniform; androconial patch distinct	4
4	Anal lobe, and occasionally part of inner margin, at UPHW with a ferruginous suffusion; UN of wings brown heavily mixed with reddish scales, surface with a distinct reddish wine color; restricted to Sierra Maestra Mountains	*Calisto smintheus*
–	Anal lobe without ferruginous suffusion at UPHW; UN of wings brown heavily mixed with pale yellow and ochre scales, surface without distinct reddish wine color; restricted to NSB Mountains	*Calisto brochei*
5	UNHW with white dot at M_2_–M_3_ no distinctly larger than remainder dots; UNHW ocellus pear shaped; UN of wings background mixed with grayish and, in less extent, pale yellow scales	*Calisto bruneri*
–	UNHW with white dot at M_2_–M_3_ distinctly larger than remainder dots; UNHW ocellus ovoid shaped; UN of wings background mixed with ochre and/or pale yellow scales	6
6	Androconial patch not entering into cell, with a rounded lobe at apex; UNHW anal lobe with a small bar of iridescent blue scales; restricted to Guaniguanico Mountains	*Calisto bradleyi*
–	Androconial patch entering into cell, apex without rounded lobe; UNHW anal lobe without small bar of iridescent blue scales; not in Guaniguanico Mountains	7
7	Male UPHW uniform dark brown, almost black; female UP of wings dark brown; area below cell at UNFW with slight red scaling; restricted to NSB Mountains	*Calisto occulta*
–	Male UPHW dark brown at basal two thirds, outer third distinctly paler; female UP of wings brown; area below cell at UNFW without slight red scaling; restricted to Guamuhaya Mountains	*Calisto muripetens*

## Discussion

The number of *Calisto* species recognized for Cuba, several more than the two accepted for most recent works (Smith et al. 1994; Lamas 2004; Sourakov and Zakharov 2011), was expected. Previous researchers, from Bates in early 1930’s to Torre in the late 1960’s, were aware of such diversity and described the majority of species, while Brown and Heineman (1972) proposed several taxonomical changes and the species number fell to only two species with a large number of subspecies.

The synonymy of all Cuban mountain species under *Calisto sibylla* was unjustified as noted before by Munroe (1972) and Núñez (2009). The absence of *Calisto sibylla* specimens for dissections and DNA sequencing left as the only means for comparisons the examination of pictures of several specimens, including the holotype, and the descriptions made by Bates (1934; 1935), which provides just wing pattern descriptions.

Despite the scarcity of evidence on hand, there are several elements pointing towards the valid species status of *Calisto sibylla*, distinct from Cuban species. The clearest difference is the lack of the reddish color in cell at UNFW, similar to only *Calisto israeli* within Cuban taxa. The presence of a black dot at both sides of HW anal lobe is also notable, being absent in all former Cuban synonyms of *Calisto sibylla*, except for *Calisto bradleyi* where the spot is edged in the UN by a small iridescent blue band. *Calisto sibylla* presents a white dot at Rs–M_1_ which is absent in *Calisto bradleyi*, *Calisto muripetens* and *Calisto occulta*. In the latter three species, the white dot at M_2_–M_3_ is distinctly larger than remainder dots whereas in all other Cuban and Bahamian species it may just be slightly larger.

The number, disposition and size of white dots at UNHW post discal area may constitute visual signals for sexual selection within *Calisto*. Robertson and Monteiro (2005) and Costanzo and Monteiro (2007) demonstrated that females of the nymphalid butterfly *Bicyclus anynana* Butler, 1879 select males based on critical features such as the size and brightness of the dorsal eyespot’s ultraviolet reflecting pupils. Several combinations of those wing pattern elements are present in Cuban *Calisto*, with sympatric species at all major mountain ranges, except perhaps Sierra Maestra, having dots located in different parts of the UNHW, varying in size and number. At NSB Mountains, *Calisto israeli* exhibits additional white reflecting elements at UNHW that probably evolved as visual signals in response to selective pressure caused by a larger number of sympatric congeners. Indeed, the existence of such a mechanism in *Calisto* needs to be tested in experiments including other reproductive isolation mechanisms like sex pheromones, probably secreted by glands associated to androconia present in males of most species.

The androconial patch at male UPFW seems to also be important in species differentiation, with the shape and conspicuousness varying between species. In all Cuban species, except *Calisto bruneri* and *Calisto herophile*, the patch is at least partially distinct from surrounding areas. In *Calisto sibylla*, as in *Calisto herophile*, the patch is hidden by the dark brown basal two thirds of FW. Such differences in the secondary sexual structure seem to constitute a key diagnostic element in *Calisto*, as noted also by Bates (1935), Michener (1943), and Johnson and Hedges (1998).

Island isolation, habitat differences and morphology suggest specific differentiation between Cuban and Bahamian *Calisto*. Whereas *Calisto sibylla* inhabits coastal thickets (Clench 1977; Harvey and Peacock 1989), all Cuban species previously regarded as synonyms to the former are found only in montane habitats.

Within the Cuban *Calisto*, genitalic characters proved to be useful in taxonomy as has been found for Hispaniolan congeners (Jonhson et al. 1987; Sourakov 1999). The most important features are the shape of digitiform projection of genitalia valve, the shape and relative size of tegumen and uncus, the relative size of female genitalia, the height of sterigmal ring dorsal crown of the latter, and the relative size of corpus bursae and ductus bursae. Previously, Bates (1934) and Torre (1968) partially illustrated and described the masculine genitalia of *Calisto smintheus* and *Calisto herophile*, as well as the uncus, gnathos and the apex of valvae of *Calisto herophile*, *Calisto bruneri*, and *Calisto smintheus*, respectively. For females, Torre (1973) poorly illustrated the genitalia of *Calisto brochei* and *Calisto israeli* without describing them; whereas Jonhson and Hedges (1998) illustrated and discussed the sterigmal ring and dorsal crown of the “*Calisto herophile* complex” and “*Calisto sibylla* complex”.

The immature stages of Cuban *Calisto* have more divergent characters than those present on adults. The case of *Calisto smintheus* and *Calisto brochei* illustrates this well. Similar to the species pair *Calisto batesi* Michener – *Calisto hysius* (Sourakov 1996) from Hispaniola, characters such as larva head capsule color pattern at all instar as well as pupae color pattern and the shape of head, last abdominal segment, and cremaster, clearly differ between species.

Preliminary DNA analyses, part of a larger work aiming to study the phylogenetic relationships of the whole genus *Calisto* and to examine their relationships with continental relatives (Matos et al. in prep.), showed that the Cuban species form a compact group. Excepting *Calisto sibylla*, which was not sequenced due to lack of fresh specimens, all species were grouped together supporting the idea of a species group: the *herophile* complex, as defined by Bates (1935) based on morphology. The average COI genetic distance supports the specific validity of all Cuban *Calisto*. Although *muripetens-occulta* and *bradleyi-herophile* species pairs exhibit relatively low values, 2.5% and 1.9% respectively, they are distinct lineages. The relationship of the *herophile* clade to Hispaniolan species remains to be tested, although Sourakov and Zakharov (2011) suggested that the Cuban species are derived from Hispaniolan species. Such a relationship with some taxa occurring on Hispaniola could be logical due to the common geological history of both islands (Pindell 1994; Iturralde–Vinent and MacPhee 1999). Furthermore, Johnson and Hedges (1998) described three species from Haiti’s Tiburon peninsula similar to Cuban species but deeper genitalic comparisons or DNA sequencing are required to confirm any possible relationship between them.

The presence of more undescribed *Calisto* species in Cuba may be expected. Ecosystems with special features like the semi desert area at extreme southeast coast or the white sand savannahs at Isla de La Juventud and Pinar del Río may still possess yet undiscovered species. Other regions are far from adequately sampled. Torre (1968) mentioned an unidentified *Calisto* specimen collected in the hills of Isla de La Juventud, the status of this entity remains unresolved. The NSB Mountains themselves are still poorly surveyed with the scarce collections in the past being focused on three or four localities mostly at foothills.

Although the phylogenetic relationships between the Cuban *Calisto* species are quite robust and well-supported, conflict between mitochondrial and nuclear datasets has been detected in *Calisto brochei*, *Calisto muripetens* and to a lesser degree in *Calisto bradleyi*. Either incomplete lineage sorting or hybridization might be invoked in those cases, as reported previously in other nymphalid genera (e.g. Bull et al. 2006; Kronforst et al. 2006; Wahlberg et al. 2009). The group *israeli*-*brochei*-*smintheus* may be a case of incomplete lineage sorting as the monophyly of *israeli* and *smintheus* are confirmed, but while the nuclear genes agree in placing *Calisto israeli* as sister to *Calisto smintheus* and *Calisto brochei*, the mitochondrial gene reconstructs the phylogeny with *Calisto israeli* as sister to all Cuban *Calisto* taxa. Similar observation has been made for the *occulta*-*muripetens*-*herophile*-*bradleyi* group, where the mitochondrial dataset infers *occulta*-*muripetens* as sister to *herophile*-*bradleyi* whereas the nuclear genes place *herophile* as sister to the *occulta*-*muripetens*-*bradleyi* clade. On the other hand, hybridization may be a common phenomenon in *Calisto* as suggested by our DNA sequence data which found several independent lineages (vouchers PM07-11, PM07-06, PM07-03 and PM15-03) that do not appear to be consistently placed within a certain clade in the tree. Based on morphology, the individual PM07-03 has been identified as *Calisto brochei* but the nuclear dataset robustly places it within *Calisto occulta*, leaving the possibility of hybridization between these two sympatric species occurring in the NSB Massif. Similarly, PM07-11 and PM07-06 may be hybrid forms of *Calisto herophile* and *Calisto bradleyi* as their phylogenetic position is not resolved and there is conflict even within the nuclear genes in placing these within either *herophile* or *bradleyi*. Clearly, a larger number of specimens needs to be analyzed genetically to discover which patterns are more common and whether this actually represents hybridization.

The origin and diversification of Cuban *Calisto* taxa remain to be studied under a rigorous biogeographic approach. However, in the present study, the phylogenetic relationships elucidated from molecular markers generate some insights about such processes. Indeed the mountains in the easternmost part of the island, including Sierra Maestra and the NSB Massif, seem to have played an important role in the diversification of the genus in Cuba, as suggested by the earlier divergence events in the phylogeny in most of the taxa occurring in those localities, whereas more derived species occupy current mountain systems in west central (*Calisto muripetens*) or western Cuba (*Calisto bradleyi*) and broad distribution ranges across the entire island (*Calisto herophile*). Although some phylogenetic relationships require further clarification, such as the *israeli*-*brochei*-*smintheus* and the *herophile*-*bradleyi*-*occulta*-*muripetens* groups, the general pattern of diversification and spreading from Sierra Maestra and NSB westwards Cuba would not be altered. Interestingly, eastern Cuba and north central Hispaniola were physically connected until the Windward Passage began to separate those landmasses by late Oligocene whereas the connection between eastern Cuba and central/western Cuba happened geologically more recently, after the disappearance of the Havana-Matanzas Channel by middle/late Miocene (Iturralde-Vinent and MacPhee 1996; Iturralde-Vinent and Macphee 1999) making it more plausible that ancestral Cuban *Calisto* taxa colonized western territories from the primitive eastern Cuba-northern Hispaniola landmass. Whether extant species are able to overcome the 80 km wide Windward Passage or not remains to be verified. Sourakov and Zakharov (2011)supported the idea of dispersal events by *Calisto* ancestors from Hispaniola to other Greater Antillean islands; however, they only included *Calisto herophile* from Cuba in their study. Future studies on the entire genus *Calisto* will allow us to assess whether the Cuban species form a monophyletic group within the Hispaniolan clade, as a sister to the Hispaniolan clade, or whether the Cuban species are in fact not a monophyletic group. What ever the case may be, a more comprehensive study will help us understand the evolutionary history of this special group of butterflies.

## Supplementary Material

XML Treatment for Calisto israeli

XML Treatment for Calisto smintheus

XML Treatment for Calisto brochei

XML Treatment for Calisto bruneri

XML Treatment for Calisto muripetens

XML Treatment for Calisto occulta

XML Treatment for Calisto bradleyi

XML Treatment for Calisto herophile
